# B cell subset distribution is altered in patients with severe periodontitis

**DOI:** 10.1371/journal.pone.0192986

**Published:** 2018-02-15

**Authors:** Julien Demoersman, Pierre Pochard, Camille Framery, Quentin Simon, Sylvie Boisramé, Assem Soueidan, Jacques-Olivier Pers

**Affiliations:** 1 UMR1227, Université de Brest, Inserm, Brest, France; 2 LabEx IGO, Brest, France; 3 Service d’odontologie, CHU Brest, Brest, France; 4 Department of Periodontology, CHU de Nantes, Nantes, France; 5 Rmes Inserm U1229/UIC11, Université de Nantes, Nantes, France; Institut Cochin, FRANCE

## Abstract

Several studies have recently highlighted the implication of B cells in physiopathogenesis of periodontal disease by showing that a B cell deficiency leads to improved periodontal parameters. However, the detailed profiles of circulating B cell subsets have not yet been investigated in patients with severe periodontitis (SP). We hypothesised that an abnormal distribution of B cell subsets could be detected in the blood of patients with severe periodontal lesions, as already reported for patients with chronic inflammatory diseases as systemic autoimmune diseases. Fifteen subjects with SP and 13 subjects without periodontitis, according to the definition proposed by the CDC periodontal disease surveillance work group, were enrolled in this pilot observational study. Two flow cytometry panels were designed to analyse the circulating B and B1 cell subset distribution in association with the RANKL expression. A significantly higher percentage of CD27^+^ memory B cells was observed in patients with SP. Among these CD27^+^ B cells, the proportion of the switched memory subset was significantly higher. At the same time, human B1 cells, which were previously associated with a regulatory function (CD20^+^CD69^-^CD43^+^CD27^+^CD11b^+^), decreased in SP patients. The RANKL expression increased in every B cell subset from the SP patients and was significantly greater in activated B cells than in the subjects without periodontitis. These preliminary results demonstrate the altered distribution of B cells in the context of severe periodontitis. Further investigations with a larger cohort of patients can elucidate if the analysis of the B cell compartment distribution can reflect the periodontal disease activity and be a reliable marker for its prognosis (clinical trial registration number: NCT02833285, B cell functions in periodontitis).

## Introduction

Periodontitis is a bacterial biofilm-induced chronic inflammatory disease leading to the destruction of tooth-supportive structures (gingiva, alveolar bone and periodontal ligament). Dysbiotic microbiota and a susceptible host are required to develop periodontitis [1], which is associated with an increased risk for certain systemic disorders such as rheumatoid arthritis, diabetes mellitus or artherosclerosis [2]. Inflammatory processes are mediated by various inflammatory and stromal cell types that lead to tissue destruction. These bacteria-induced inflammatory mechanisms are the suspected links between periodontitis and inflammatory systemic syndromes [3,4]. Despite a better management of periodontitis, the prevalence of severe periodontitis (SP) remained stable for thirty years [5]. Diagnosis and monitoring of SP rely on traditional clinical examinations which are inadequate to predict patient susceptibility, disease activity, and response to treatment [6]. The requirement for reliable biomarkers to distinguish progressive periodontitis from normal biological processes is considered fundamental to conduct the appropriate treatment.

Despite their high predominance in advanced periodontal lesions [7,8], B cell and plasma cell functions in periodontitis remain incompletely characterised. B cells seem to have a dual role in periodontitis, both protective by facilitating bacterial clearance and destructive by promoting inflammation, bone resorption and matrix dissolution [9,10]. In this context, B cells produce not only a variety of anti-inflammatory cytokines, such as IL-10 and tumor growth factor (TGF)-β, but also pro-inflammatory factors, such as tumour necrosis factor (TNF)-α, interleukin (IL)-6 or matrix metalloproteinases, which contribute to the degradation of connective tissue. Regulatory B cells, which are deficient in some autoimmune diseases, can also have a role in periodontitis [11]. Regulatory B cells are indeed a source of anti-inflammatory cytokines (e.g. IL-10 and TGF-β), express high levels of CD25 and CD86, and are able to suppress Th1 proliferation and contribute to the maintenance of self-tolerance [11].

Bone resorption is mediated by the triad receptor activator of nuclear factor ĸB ligand (RANKL)/osteoprotegerin (OPG)/RANK. RANKL is a ligand for RANK, a receptor expressed by osteoclast precursors, and a RANK-RANKL interaction promotes osteoclastogenesis [12]. Interestingly, B cells have been reported to be a major source of RANKL in periodontitis [13].

As the important role of B cells in physiopathogenesis of periodontal disease has been recently highlighted by studies showing that a B cell deficiency leads to improved periodontal parameters [14–17], we hypothesised that an abnormal distribution of B cell subsets could be detected in the blood of patients with severe periodontal lesions, as already reported for patients with chronic inflammatory diseases as systemic autoimmune diseases. We also assessed the RANKL expression in the B cell subsets in connection with the severity of periodontitis. This pilot study showed an increase in memory and activated B cells expressing RANKL and a depletion of circulating CD11b^+^ B1 cells with a regulatory phenotype in SP patients.

## Materials and methods

### Subject recruitment

**Fig 1:**
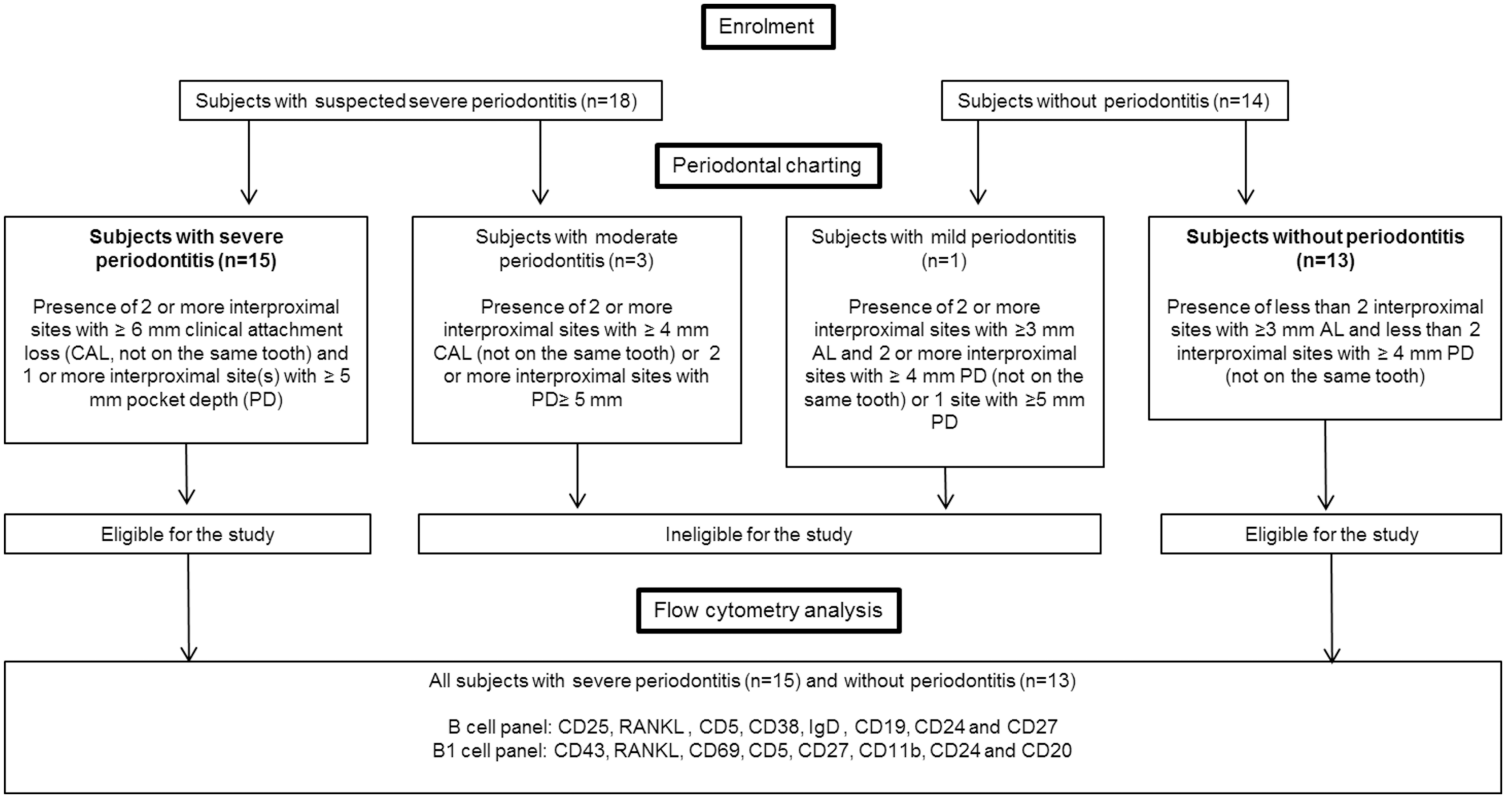
STROBE checklist.

This publication is conformed to the STROBE guidelines (Fig 1 and S1 Table). This study was conducted in a dental clinic at Brest Hospital in France. The protocol was registered on clinicaltrials.gov (NCT02833285) (S1 and S2 Figs) and approved by the ethical committee of the Brest hospital (April 9th, 2015) (S3 Fig). Between May 2015 and December 2016, 28 subjects were recruited and included in two different groups, namely, 15 subjects with SP and 13 subjects without periodontitis [non-periodontitis (NoP) control group], according to the definition proposed by the CDC periodontal disease surveillance work group and supported by the American Academy of Periodontology to define periodontitis severity [18]. SP was defined as the presence of two or more interproximal sites with ≥ 6 mm clinical attachment loss (CAL, not on the same tooth) and one or more interproximal site(s) with ≥ 5 mm pocket depth (PD). According to the same classification, patients with moderate periodontitis (those who did not meet the SP case definition, and defined as 2 or more interproximal sites with ≥ 4 mm CAL (not on the same tooth) or 2 or more interproximal sites with PD≥ 5 mm, also not on the same tooth) or mild periodontitis (those who met neither the severe nor moderate periodontitis case definition, defined as ≥2 interproximal sites with ≥3 mm AL and ≥2 interproximal sites with ≥ 4 mm PD (not on the same tooth) or 1 site with ≥5 mm PD) were excluded from this study. Consequently, the second group is only composed of patients without periodontitis.

The exclusion criteria were: minor, patient having taken antibiotics in the previous 3 months, patients with systemic diseases including chronic inflammatory disease, pregnancy, orthodontic treatment ongoing. Furthermore all the enrolled patients and controls had no treatment with corticosteroids, immunosuppressants or biological agents. A consent chart to participate in the study was signed, and the samples were managed and recorded in a biologic collection of human samples at Brest Hospital (OdontoBrest biocollection: DC 2014–2158).

### Clinical measurements

The same examiner (CF) routinely performed all clinical examinations and periodontal charting. The whole-mouth clinical periodontal parameters, including O’Leary plaque index, bleeding on probing (BOP), CAL and PD, were determined at four interproximal sites per tooth. PD was defined as the distance in millimetre (mm) from the most coronal portion of the free gingival margin to the most apical penetration of the periodontal probe. CAL was defined as the distance in mm from the cemento–enamel junction to the most apical penetration of the probe. PD and CAL were measured with a constant pressure periodontal probe and recorded to the nearest mm; every observation close to 0.5 mm was rounded off to the lower whole number. Decayed, missing and filled teeth, oral hygiene habits, smoking status and removable or fixed prosthetic appliances were assessed.

All periodontal chart measurements were noted in a programmed Excel table (Microsoft, 2011). Clinical indices, periodontal scores and diagnosis according to the Page and Eke classification [18] were automatically calculated for each subject.

### Flow cytometry analysis of circulating B cells

Peripheral blood was collected for biological and cytometry analyses. Blood was analysed in two panels dedicated to B cells. For the first panel, 200 μl of blood was previously washed twice with phosphate-buffered saline to remove free IgM. This nine-colour panel includes FITC-conjugated anti-CD25, PE-conjugated anti-RANKL (Biolegend), ECD-conjugated anti-CD5, PC5.5-conjugated anti-CD38, PC7-conjugated anti-CD27, APC-conjugated anti-IgD (Becton Dickinson), AAF700-conjugated anti-CD19, AAF750-conjugated anti-CD24 and pacific blue-conjugated anti-IgM antibodies (Abs). This panel distinguished CD25^+^ activated CD19^+^ B cells, CD19^lo^CD38^hi^CD27^hi^ plasmablasts, CD19^+^CD38^hi^CD24^hi^ transitional B cells, CD19^+^CD5^+^ B cells, switched memory CD19^+^IgD^-^CD27^+^ B cells, unswitched memory CD19^+^IgD^+^CD27^+^ B cells, double negative CD19^+^IgD^-^CD27^-^ B cells, CD19^+^IgD^+^CD27^-^CD38^+^CD24^lo^ mature B cells and CD19^+^IgD^+^CD27^-^CD38^-^CD24^+^ naïve B cells. The second eight-colour panel was designed for a fine analysis of CD5^+^ B cells and human B1 cells as described by Griffin et al [19]. This panel included FITC-conjugated anti-CD43, PE-conjugated anti-RANKL, ECD-conjugated anti-CD69, PC5.5-conjugated anti-CD5, PC7-conjugated anti-CD27, APC-conjugated anti-CD11b, AAF750-conjugated anti-CD24 and pacific blue-conjugated anti-CD20 Abs. This panel distinguished CD20^+^CD11b^+^CD5^+^, CD20^+^CD11b^+^CD5^-^, CD20^+^CD11b^-^CD5^+^ B cells and B1 cells defined as CD20^+^CD69^-^CD43^+^CD27^+^. All Abs unless specified were from Beckman Coulter. Red blood cells were lysed using Versalyse^®^ solution (Beckman Coulter, Fullerton, CA) before analysis on the Navios flow cytometer (Beckman Coulter).

### Statistical analysis

Statistical analyses were performed by GraphPad (Prism IBM SPSS, GraphPad 5). Non-parametric variables were analysed by the Mann–Whitney or Fisher test and correlated by the Pearson r test. A P value less than 0.05 was considered statistically significant.

## Results

### Distribution of total leukocytes is normal in severe periodontitis

Among the 15 patients included in the SP group, six were women (39%) compared with seven (54%) in the NoP control group (p = 0.7). The mean age was 42.1±10.0 for SP patients and 30.8±9.7 for the NoP controls (p = 0.0023). Smoking habits were 32% for SP patients and 38% for NoP controls (p = 1). In whole blood, patients with SP have the same absolute number of leukocytes with the NoP controls (6658±2159 *vs*. 5614±1185, respectively, p = 0.3). No difference was observed in the distribution and absolute number of total B cells between the SP and NoP groups (9.0±3.5% *vs*. 8.3±2.9%, p = 0.40 and 310±234 *vs*. 105±110, p = 0.11 respectively).

### Memory B cells are predominant in patients with severe periodontitis

**Fig 2:**
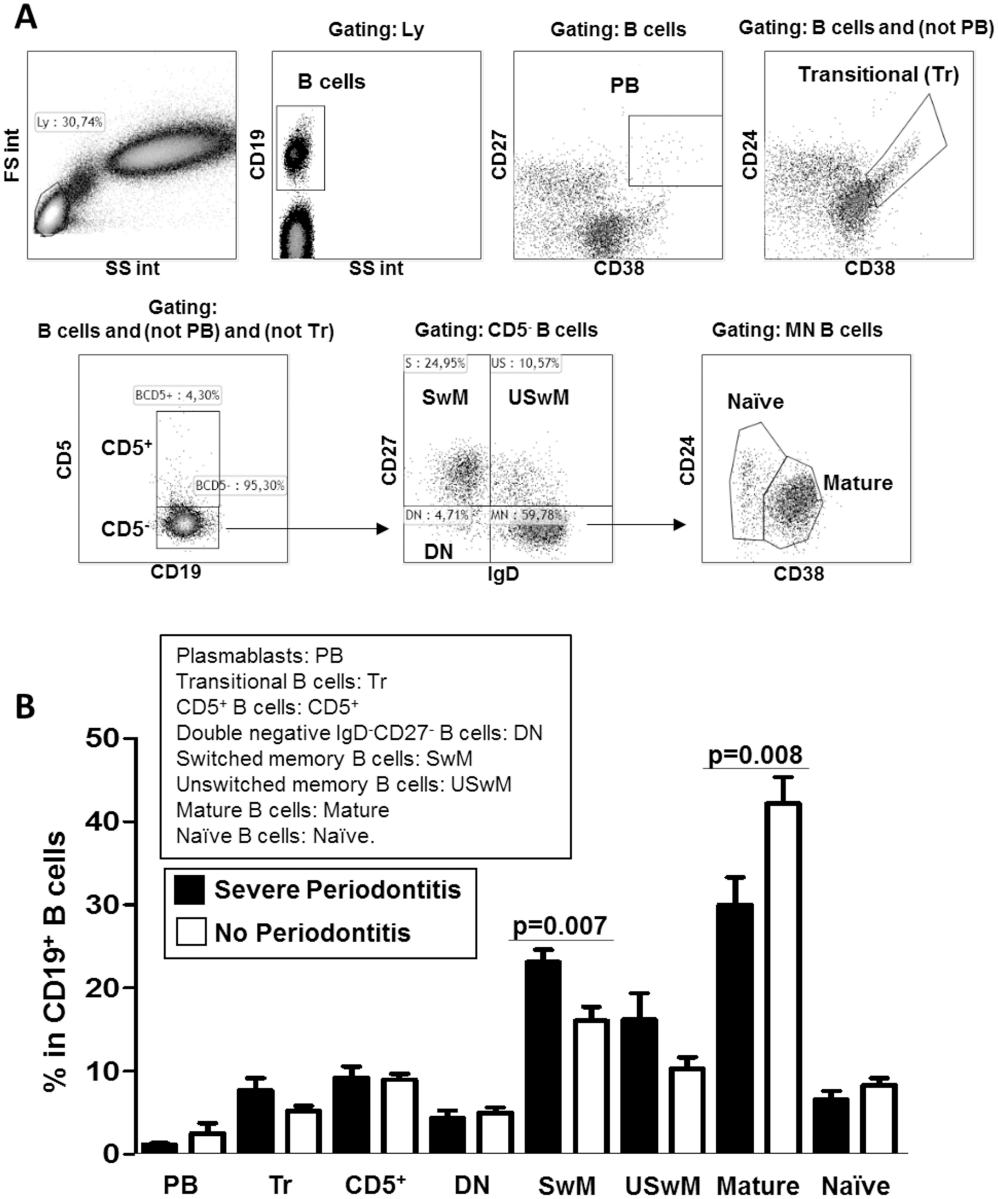
Phenotype of B subsets in whole blood of patients with severe periodontitis (SP) and controls (No Periodontitis). **A**) The gating strategy used after doublet exclusion allows the characterisation eight different and distinct B cell subsets. **B**) Circulating B cell distribution reveals an increase in the switched memory B cells and a decrease in the mature B cells in the SP patients in comparison with the controls. p value is indicated when significant. Plasmablasts: PB; Transitional B cells: TR; CD5^+^ B cells: CD5^+^; double negative IgD^-^CD27^-^ B cells: DN; switched memory B cells: SwM; unswitched memory B cells: USwM; mature B cells: Mature and naïve B cells: Naïve.

A fine analysis of the B cell subset distribution was conducted according to the gating strategy described in Fig 2A. Changes in the B cell subset distribution were then observed in the SP patients with a notably higher percentage and absolute number of CD27^+^ memory B cells (42.7±11.6% for SP patients *vs*. 29.4±11.3% for NoP controls, p = 0.01, and 151±137 for SP patients *vs*. 42±12 for NoP controls, p = 0.046). Among these CD27^+^ B cells (Fig 2B), the proportion in B cells and the absolute number of the switched memory subset (CD27^+^IgD^-^IgM^-^) were significantly higher in the SP patients in comparison with the NoP controls (23.1±8.0% *vs*. 16.0±6.0%, p = 0.007, and 66±52 *vs*. 25±10, p = 0.024, respectively). Interestingly, the percentage of mature B cells decreased among the SP patients (30.0±12.7% for SP patients *vs*. 42.1±11.5% for the NoP controls, p = 0.008), thus suggesting a B cell differentiation in the memory phenotype among the SP patients (Fig 2B).

### B1 cells and CD11b^+^CD5^+^ B cells are reduced in severe periodontitis patients

**Fig 3:**
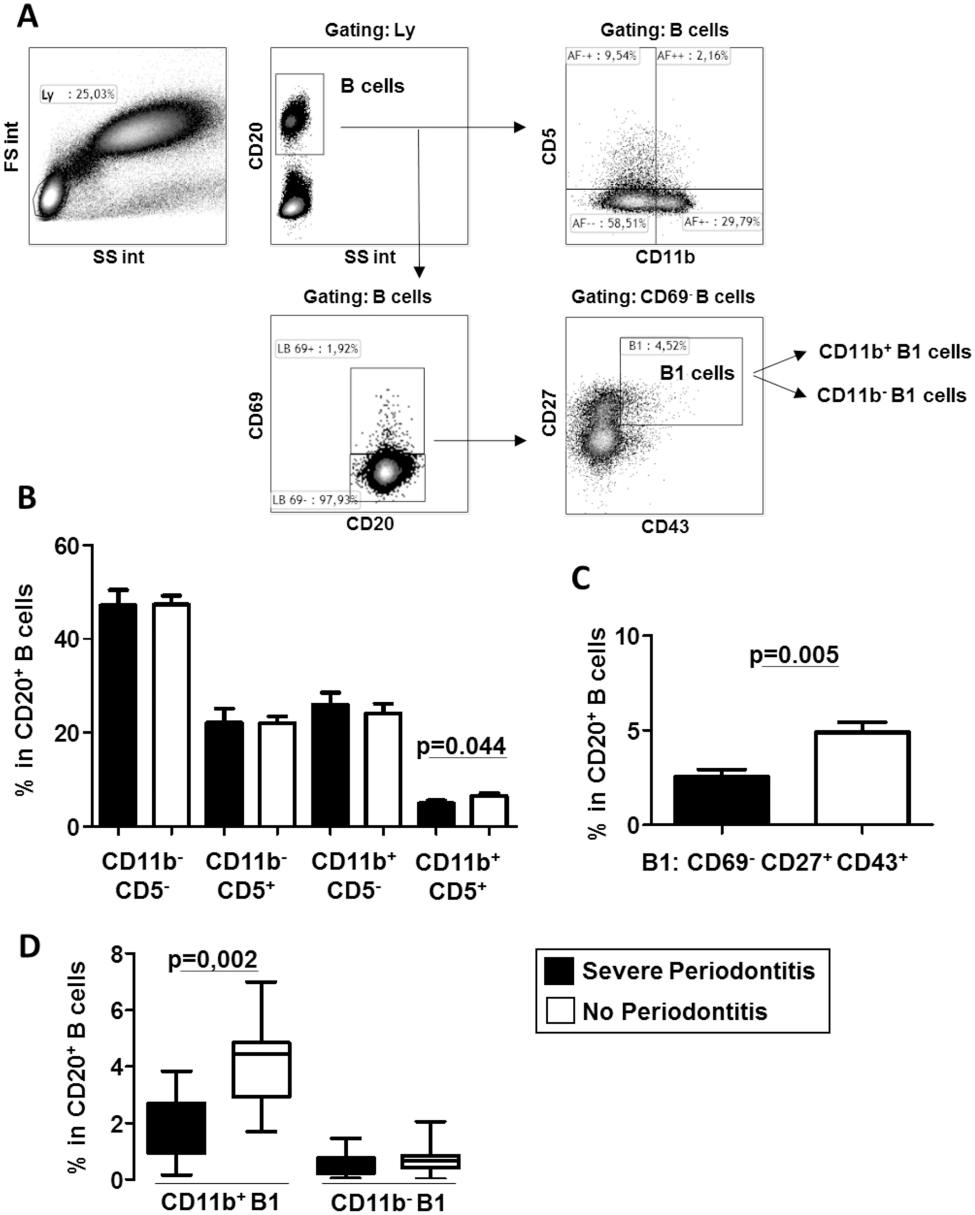
Phenotype of B1 and CD5^+^ B cells in whole blood of patients with severe periodontitis (SP) and controls (No Periodontitis). **A**) The gating strategy used after doublet exclusion allows the characterisation of B cells according to CD11b and CD5 expression but also the human B1 subset (CD20^+^CD69^-^CD43^+^CD27^+^). **B**) Circulating B cell distribution according to CD11b and CD5 expression reveals a decrease in the CD11b^+^CD5^+^ B cells in the SP patients in comparison with the controls. **C**) B1 B cells are lower in the SP patients in comparison with the controls. **D**) Among B1 cells the CD11b^+^ B1 regulatory subset is markedly decrease in the SP patients. p value is indicated when significant.

Although the CD5^+^ B cell distribution appeared similar the SP group and the controls (9.2±5.3% for SP patients *vs*. 9.0±2.6% for NoP controls, Fig 2B), we analysed this subset previously reported to be higher in gingival tissue of periodontitis patients [7, 20]. The gating strategy is described in Fig 3A. The proportion of CD20^+^CD11b^+^CD5^+^ B cells, previously associated with a regulatory phenotype, was lower in SP patients than in the NoP controls (5.0±2.3% *vs* 6.5±2.1%, p = 0.044, respectively, Fig 3B). Interestingly, a decrease in the B1 CD20^+^CD69^-^CD43^+^CD27^+^ subset was observed in the SP patients in comparison with the controls (2.5±1.5% *vs*. 4.9±1.9%, p = 0.005, respectively, Fig 3C). Phenotypic and functional subdivisions of human B1 cells have been described: the CD11b^-^ B1 cells primarily secrete antibody, and the CD11b^+^ B1 cells spontaneously produce IL-10 and suppress T-cell activation [21]. We reported an important decrease in CD11b^+^ B1 cells in the SP patients in comparison with the controls (2.0±1.1% for SP patients *vs*. 4.2±1.6% for the NoP controls, p = 0.002). The CD11b^-^ B1 subset was not different between the two groups (0.5±0.4% for SP patients *vs*. 0.7±0.5% for the NoP controls). Altogether, these results suggest a depletion of circulating B cells with a regulatory phenotype in SP patients in comparison with the NoP control group.

### RANKL expression in B cell subsets

**Fig 4:**
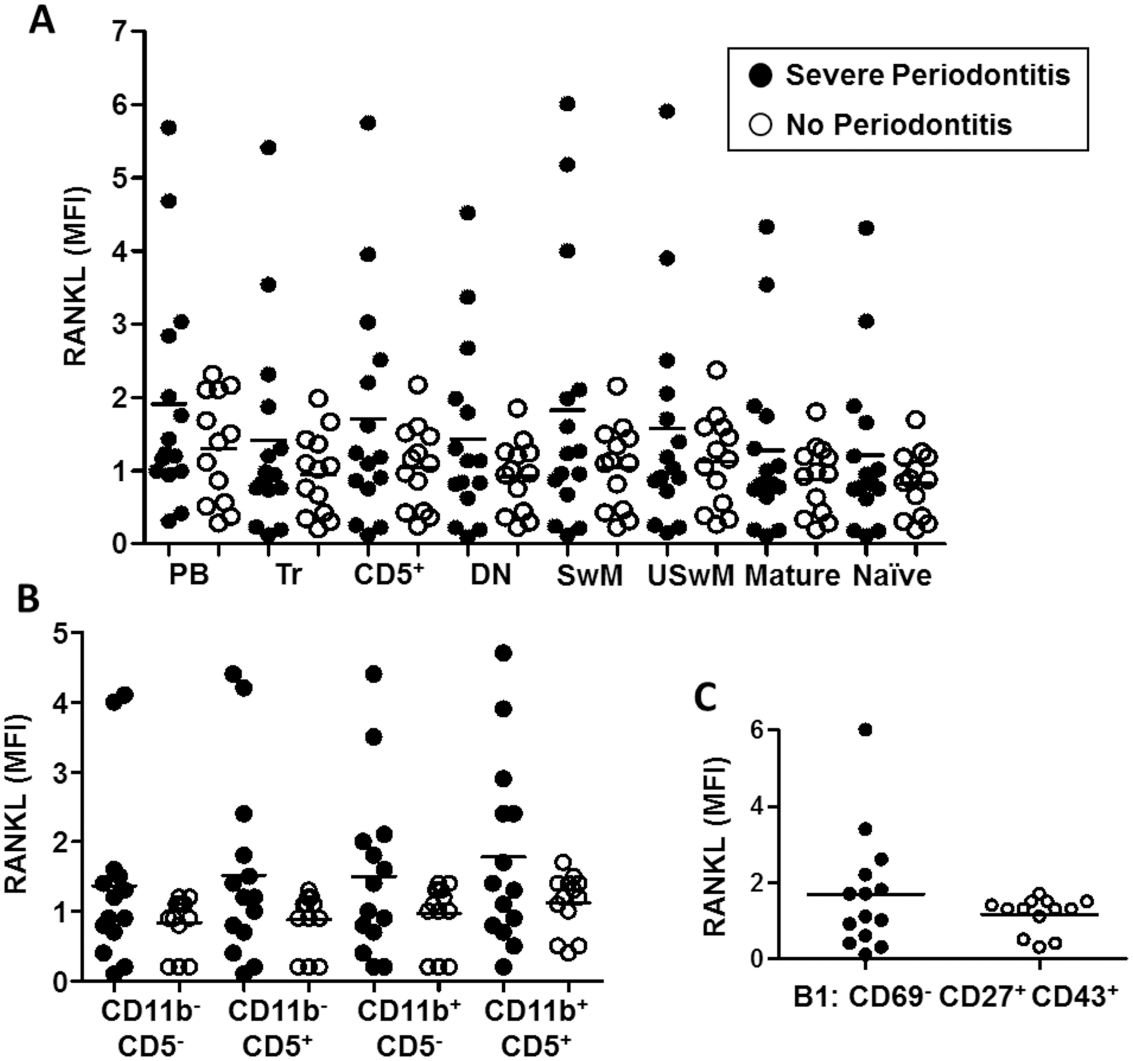
Expression of RANKL in the B cell subsets from patients with severe periodontitis and controls (No Periodontitis). **A)** Expression of RANKL in the eight distinct B cell subsets defined in Fig 1. **B)** Expression of RANKL in the circulating B cell distribution according to CD11b and CD5 expression. **C**) Expression of RANKL in B1 cells. Mean fluorescence intensity: MFI; Plasmablasts: PB; Transitional B cells: TR; CD5^+^ B cells: CD5^+^; double negative IgD^-^CD27^-^ B cells: DN; switched memory B cells: SwM; unswitched memory B cells: USwM; mature B cells: Mature and naïve B cells: Naïve.

The RANKL expression in B cell subsets was assessed in all B cell subsets (Fig 4). The RANKL mean fluorescence intensity (MFI), which corresponds to the level of RANKL expression in the membrane of B cells, appears to be important in some patients with SP while never increased in the NoP group. When increased, the RANKL MFI on B cells in SP patients (Fig 4) was not specific to a cell subset but appeared to be related to a general increase in expression in all subsets.

### B cells from severe periodontitis patients present an activated phenotype

**Fig 5:**
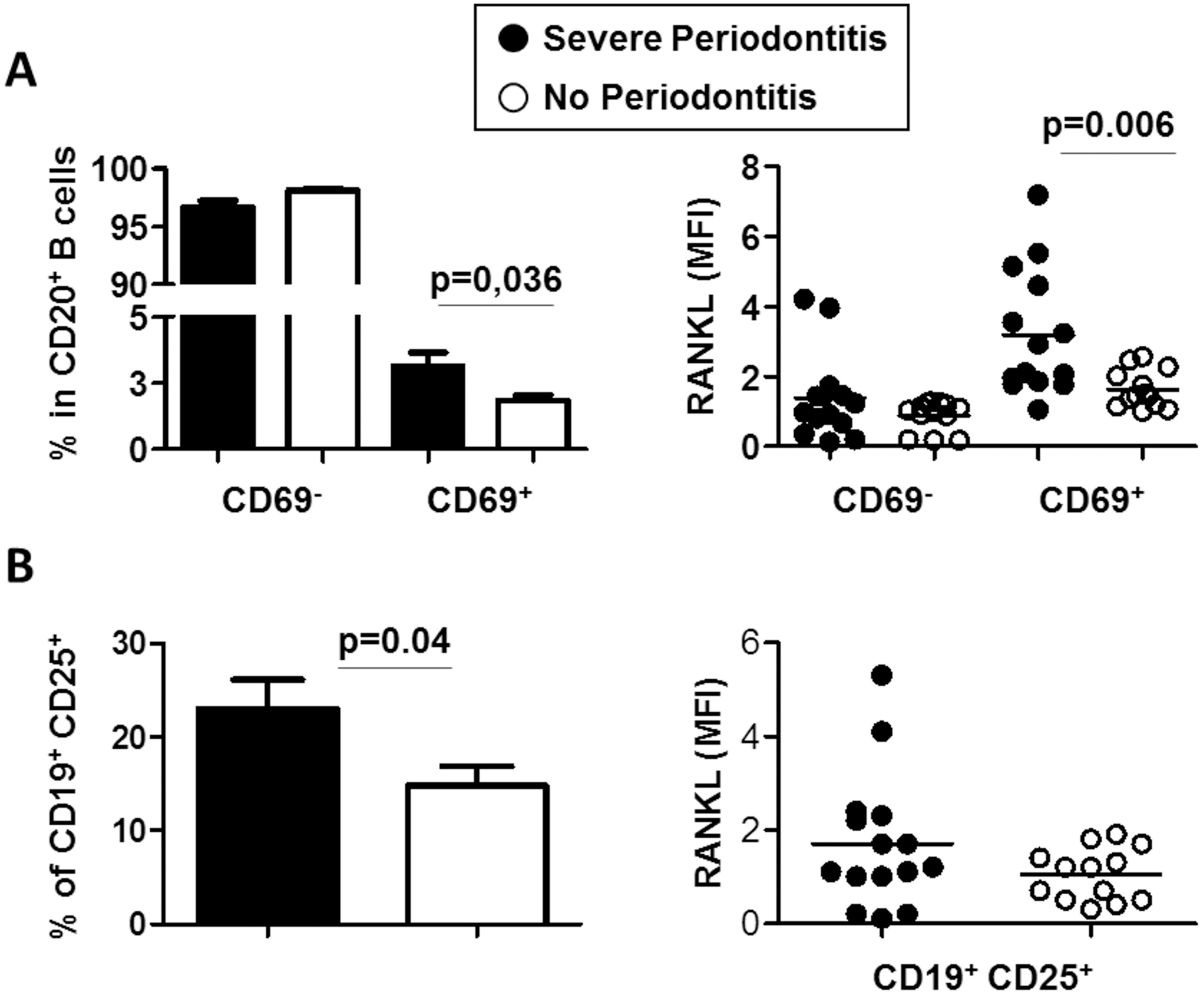
Expression of B cell activation markers in the B cell subsets from patients with severe periodontitis (SP) and controls (No Periodontitis). **A**) The percentage of CD69^+^ B cells expressing RANKL was higher in the SP patients in comparison with the controls. **B**) The percentage of CD25^+^ B cells was higher in the SP patients in comparison with the controls. Mean fluorescence intensity: MFI, p value is indicated when significant.

CD69 and CD25 were described as B cell activation markers. The percentages and absolute numbers of CD69^+^ (Fig 5A) and CD25^+^ (Fig 5B) B cells were higher in the SP patients in comparison with the controls (3.1±2.0% *vs*. 1.8±0.7%, p = 0.036, and 11±8 *vs*. 3±1, p = 0.006 for CD69^+^ B cells and 23.6±11.9% *vs*. 15.6±6.9%, p = 0.004, and 77±79 *vs*. 20±9, p = 0.014, for CD25^+^ B cells, respectively). The RANKL MFI significantly increased in CD69^+^ activated B cells from SP patients in comparison with that from the NoP controls (3.3±1.7 *vs*. 1.6±0.5, p = 0.006, respectively, Fig 5A).

## Discussion

Our pilot study showed for the first time that SP patients presented an altered distribution of peripheral blood B cells with a higher proportion of memory B cells, especially switched memory B cells, and a lower proportion of regulatory CD11b^+^ B1 cells.

A limitation of this study could be the age difference between the SP and the non-periodontitis groups. Some studies [22,23] have described, in the elderly, a reduction of naïve B cells associated with the expansion of memory B cells. However, these memory B cells showed a senescence-associated phenotype with the accumulation of a CD27^-^IgD^-^ double negative population. More interestingly, the switched memory B cells, significantly higher in SP patients in our study, were reported lower both in frequency and absolute number with age, suggesting an intrinsic defect in the ability of such B cells from elderly individuals to undergo class switch [24]. Furthermore, although the proportion of CD19^+^ B cells decreases until age 35, B cells remains steady up to age 65 [25] and when young healthy donors (HD) (mean age = 28.5±1.9 years) and mild-aged HD (mean age = 69.1±3.9 years) are compared, similar percentages of CD27^+^IgD^-^ switched memory B cells were found [26]. The same observation was reported by Morbach et al [27], with reference values for memory B cells, in 26 to 50 years old HD, corresponding to 15.2% (13.4–21.4) for CD27^+^IgD^+^ unswitched memory and to 13.2% (9.2–18.9) for CD27^+^IgD^-^ switched memory B cells. These reference values are similar to those observed in our NoP control group. In healthy adults, B cells represent 9–10% of all lymphocytes [27]. The proportions of B cells, both in the periodontitis and non-periodontitis groups, are consistent with this description. Therefore, SP does not seem to affect the number of circulating B cells. When comparing healthy controls with patient with periodontal disease stages (gingivitis and moderate periodontitis), Mahanonda et al. observed no differences in the peripheral blood B cell subsets [28]. In this study, the different subsets of B cells were only identified employing a mixture of 3 mAbs (anti-CD19, anti-CD27 and anti-CD38 mAbs) when compared to the 9-colour panel used in our study. Consequently, memory B cells were characterized as CD19^+^CD27^+^CD38^-^ and they cannot distinguish switched memory B cells from unswitched memory B cells. However, they found that memory B cells (CD19^+^CD27^+^CD38^-^) represented the majority in the B cell population in clinically healthy gingiva and in gingivitis tissues (around 90% of the B cell population). The density of memory B cells was lower in periodontitis tissues (around 40% of the B cell population), and plasma cells were predominant. Interestingly, in our study, memory B cells were higher in the peripheral blood of SP patients, but mature B cells were lower in comparison with the NoP group. Furthermore, the distribution of naïve and transitional B cell subsets was equivalent in both groups. These observations suggest a B cell differentiation process that occurs in the secondary lymph nodes of SP patients. We can then hypothesise that memory B cells will reach gingival tissues, where a terminal differentiation into plasmablasts and plasma cells takes place.

A strong expression of CXCL13 in the endothelium was observed in periodontal tissues, suggesting that CXCL13 played a role in B cell entry to gingival tissues [29]. Furthermore, BAFF mRNA and protein were upregulated in gingival tissues from both patients with chronic periodontitis and those with experimental periodontitis, and the augmentations correlated with the increased numbers of B cells/plasma cells [30]. In this context, the BAFF-mediated increase in CXCL13-dependent chemotaxis was reported to be greater for the memory B cells than for the naive B cells [31] regulating the memory B cell recruitment in the gingival tissues of SP patients.

During their development and maturation through the germinal centre, memory B cells are somatically mutated in class-switched memory B cells, thus losing their IgD expression and replacing IgM by another immunoglobulin (IgA, IgG or IgE) [32]. Our results showed a predominance of switched memory B cells in SP, and the development of switched memory B cells was intimately connected with plasma cells development [32]. Our preliminary findings on cell distribution in gingival biopsies from SP patients by flow cytometry analyses after elution confirmed the importance of the plasmablast compartment [7,20].

Interestingly, periodontal therapy resulted in a reversal shift of the tissue B cell profile, which became comparable with the one observed in clinically healthy and gingivitis tissues, where the majority of B cells were memory and not plasma cells [28]. Consequently, evaluating whether the level of peripheral memory B cells returns to normal after periodontal treatment and could be used as a good prognostic marker for patients is important in future studies. This abnormal shift can then be used in the follow-up of patients.

However, some questions remain regarding the role of these memory B cells in the peripheral blood of SP patients. What is the antigen specificity of these memory B cells? Do they target periodontal bacteria in the context of chronic inflammation? Do these memory B cells differentiate into antibody-secreting plasma cells? Do these peripheral blood memory B cells emigrate from the gingival tissue, where they have been educated, or are these cells in transit?

Another important observation is the association between the higher level of memory B cells in the peripheral blood and the severity of periodontitis. Relapse after B cell-depleted therapy correlating with the preferential reconstitution of memory B cells was previously observed in autoimmune diseases, such as systemic lupus erythematosus (SLE) and rheumatoid arthritis [33–35]. When compared with that of several autoimmune diseases, the percentage of CD27^+^ memory B cells remained higher in SP (42.7±11.6% for SP patients, 29.4±11.3% for NoP controls, 29.9±19.8% for patients with rheumatoid arthritis (n = 52), 24.9±19.9% for patients with primary Sjögren’s syndrome (n = 48) and 40.2± 21.8% for patients with SLE (n = 50), personal data). Consequently, further examination of this memory compartment can yield informative metrics of the disease. To some extent, the periodontal status should be evaluated in patients with autoimmune diseases.

Another B cell subset previously reported in autoimmune diseases [36] because of its association with the production of autoantibodies was identified in periodontitis. This B cell subset referred as to autoreactive CD5^+^ B cells was inconsistently reported in periodontitis patients. Whereas some groups [37,38] reported large numbers of CD5^+^ B cells in the peripheral blood of patients with advanced periodontitis compared with the controls, others [39] found no difference.

All these studies characterise CD5^+^ B cells by the sole expression of CD5. However, in mice, these autoreactive B cells, also called B1, were further subdivided into B-1a (CD11b^+^CD5^+^) and B-1b (CD11b^+^CD5^-^) cells [40], which are developmentally, phenotypically and functionally distinct, but are localised in similar anatomical sites as the pleural and peritoneal cavities. Recently, Griffin et al. found that a small subset of human peripheral CD20^+^ B cells expressing CD43 and CD27 recapitulated the key functional characteristics of murine B1 cells [19] and demonstrated that only the CD11b^-^ B1 cells in humans primarily secrete antibody and the CD11b^+^ B1 cells spontaneously produce IL-10 and suppress T-cell activation [21]. Consequently, the latter B1 subset could be considered as regulatory B cells. We reported here a large decrease in the CD11b^+^ B cell subset in SP patients. Interestingly, two recent studies demonstrated that IL-10-producing regulatory B cells, when transferred in mice, reduced periodontal bone loss [41,42]. Then, a decrease in regulatory CD11b^+^ B1 cells could skew the immune response into an aggravating progression of periodontal disease. Consequently, similar to the abnormal distribution of switched memory B cells, CD11b^+^ B1 cell distribution could be monitored in SP patients and used as a key marker of the disease status.

B cells express RANKL, a key molecule involved in osteoimmunology and bone resorption in periodontitis [13,43]. However, only some individuals express RANKL in all B cell subsets while others lack RANKL expression in all B cell subsets. Consequently, the variability in RANKL expression between individual patients deserves further investigations. RANKL is also known for its role in controlling the plasticity of the immune system and secondary lymph node organization [44,45]. B cells appear to be an active regulator of the RANK/RANKL/OPG axis [43], and activated B cells promote osteoclastogenesis [46]. Interestingly, we observed that activated CD25^+^ and CD69^+^ B cells were predominant in SP in comparison with the NoP controls. The activated B cells express higher levels of RANKL. Therefore, the composition of lymphocyte subpopulations is crucial for osteoclastogenesis in periodontitis. Moreover, anti-B cells therapy leads to a reduction of bone resorption in RA and seems to be beneficial in improving periodontitis [14]. In RA, activation through BCR and CD40 induces switched memory B cells to express RANKL and leads to the activation of osteoclastogenesis [47]. A similar mechanism could be suspected in periodontitis and could explain the general increase in the observed RANKL expression on B cells in our study. Indeed, B cells have been reported as the main source of RANKL in periodontitis [13].

This pilot study highlights that SP is associated with an increase in memory B cells in peripheral blood and that this association could define a new signature of SP. Nevertheless, further investigations with a larger cohort of patients could elucidate if the analysis of the memory B cell compartment distribution could reflect the disease activity and be a reliable marker for its prognosis. This information, in connection with the percentage of circulating B1 cells, could define an interesting complementary marker that could be analysed (ratio of memory B cells to B1 cells) before and after the treatment to check the response to treatment and disease evolution.

## Supporting information

S1 Table This is the STROBE_checklist. (DOCX)

S1 Fig This is the complete and detailed plan for the conduct and analysis of the LBPARO trial that the ethics committee approved before the trial began (in French). (PDF)

S2 Fig This is a translation in English of the trial as described in clinicaltrial.gov website. (PDF)

S3 Fig This is the ethics committee review board approval of the study (in French). (PDF)
