# Supplementary material for: B cell subset distribution is altered in patients with severe periodontitis
Source: PLoS One. 2018 Feb 15;13(2):e0192986. doi: 10.1371/journal.pone.0192986 (PMC5814041; doi:10.1371/journal.pone.0192986)
Supplement: S1 Fig — (PDF) [file pone.0192986.s002.pdf]

PARODONTITE ET INFLAMMATION : APPROCHE CLINIQUE ET BIOLOGIQUE DU RÔLE DU LYMPHOCYTE B.

LBPARO

Version V1.0 du 17/04/15

➢ Responsable de la recherche :

Pr. Jacques-Olivier PERS - CHRU de Brest, Hôpital Morvan – Laboratoire d'Immunologie – 2 av Foch – 29609 Brest Cedex
Tel : 02 98 32 33 84
Mail : pers@univ-brest.fr

➢ Investigateurs associés
Cliniciens dans le service d'odontologie du CHRU de Brest
Camille FRAMERY
Dr Juliette COAT
Dr Claire HENRY
Dr Guy LETOUX
Dr Sylvie BOISRAME-GASTRIN
Dr Cédric LANSONNEUR

➢ Scientifiques associés
Personnel du laboratoire d'Immunologie
Dr Julien DEMOERSMAN
Dr Pierre POCHARD
Dr Laetitia LE POTTIER
Quentin SIMON

➢Statisticien

Pr Jacques Olivier PERS

SOMMAIRE

1. RESUME

2. JUSTIFICATION SCIENTIFIQUE ET DESCRIPTION GENERALE DE LA RECHERCHE.

3. OBJECTIF

4. CONCEPTION ET DEROULEMENT DE LA RECHERCHE

5. SELECTION ET EXCLUSION DES PERSONNES DE LA RECHERCHE

6. MODALITES DE RECRUTEMENT ET D'INFORMATION DES PERSONNES CONCERNEES

7. STATISTIQUES

8. TRAITEMENT DES DONNEES ET CONSERVATION DES DOCUMENTS ET DES DONNEES RELATIVES A LA RECHERCHE

9. FINANCEMENT

10. PUBLICATION

11. BIBLIOGRAPHIE

12. ANNEXES

Informations générales

PARODONTITE ET INFLAMMATION : APPROCHE CLINIQUE ET BIOLOGIQUE DU ROLE DU LYMPHOCYTE B.

- ***Pr Jacques Olivier PERS / Dr Julien DEMOERSMAN***

- Dr Juliette COAT
- Dr Claire HENRY
- Dr Guy LETOUX
- Dr Sylvie BOISRAME-GASTRIN
- Dr Cédric LANSONNEUR
- Camille FRAMERY

- ***Coordination et suivi de l'étude***

- Pr Jacques Olivier Pers

- ***Scientifiques associés***

- Dr Julien DEMOERSMAN
- Dr Laetitia LE POTTIER
- Dr Pierre POCHARD
- Quentin SIMON

Plateau technique associé

1. RESUME

TITRE	Parodontite et inflammation : approche clinique et biologique du rôle du lymphocyte B.
INVESTIGATEUR RESPONSABLE	Pr J-O Pers
ACRONYME	LBParo
JUSTIFICATION	Le lymphocyte B est un acteur majeur dans la mise en place et l'évolution des maladies parodontales.
POPULATION CONCERNEE	Cas : Patients souffrant de maladie parodontale chronique ou agressive.<br>Témoins : Patients ne souffrant pas de parodontite et devant subir une chirurgie dentaire incluant la réalisation d'une analyse sanguine.
OBJECTIF PRINCIPAL	Analyse du phénotype des lymphocytes B au niveau du sang et de la gencive des patients souffrant de différents types de maladies parodontales
OBJECTIFS SECONDAIRES	Evaluation des effets des traitements parodontaux sur le phénotype et la fonction des lymphocytes B<br>Etude du profil d'expression de l'agressivité des maladies parodontales par l'analyse en cytométrie en flux
CRITERE D'EVALUATION PRINCIPAL	Analyse Phénotypique des lymphocytes B dans le sang et les gencives
CRITERES D'EVALUATION SECONDAIRES	Immunofluorescence sur échantillons de gencive<br>Analyse des constituants circulants du plasma<br>Caractérisation et mesure de différentes cytokines présentent au niveau du fluide gingival<br>Analyse microbiologique du biofilm sous-gingival
METHODOLOGIE	Etude physiopathologique prospective monocentrique sur données clinocobiologiques<br>Analyse sur plusieurs groupes de parodontite en fonction de critères cliniques (agressive, chronique) et d'activité vs groupe contrôle sans parodontite
STATISTIQUE	Comparaison de groupe avec variables quantitatives et qualitatives<br>Tests t de student non appariés pour les variables quantitatives paramétriques<br>Test U de Mann-Withney pour les variables quantitatives non paramétriques<br>Test de Khi-2 ou de Fisher pour les variables qualitatives<br>Test ANOVA ou de Kruskal Wallis pour la comparaison des groupes
CRITERE D'INCLUSION	<u>Cas :</u><br>- Homme ou femme de 18 à 75 ans avant traitement parodontal<br>- Souffrant d'une parodontite chronique ou agressive,

	- Nécessitant une chirurgie type parodontale<br>- Avec une suspicion de troubles de l'hémostase ou de la présence d'un diabète<br>- En bonne santé général<br>- Ayant signé leur consentement<br><br><u>Témoins :</u><br>Sujets majeur, sans parodontite et devant subir une extraction dentaire ou une chirurgie mucogingivale avec suspicion de troubles de l'hémostase ou la présence de diabète, en bonne santé général et ayant donné son consentement.
CRITERES DE NON-INCLUSION	<u>Cas :</u><br>- Mineur<br>- Patient ayant pris des antibiotiques dans les 3 mois précédents l'étude<br>- Patient présentant une pathologie systémique notamment pathologie inflammatoire chronique<br>- Femme enceinte<br>- En cours de traitement orthodontique<br><br><u>Témoins:</u><br>- Mineur<br>- Souffrant de parodontite<br>- Prise d'antibiotiques dans les 3 mois qui précèdent l'étude<br>- Femme enceinte<br>- Pathologie systémique, notamment une pathologie inflammatoire chronique<br>- En cours de traitement orthodontique
NOMBRE DE PATIENTS	15 patients avec une parodontite chronique 15 patients avec une parodontite agressive, 30 témoins.
CALENDRIER	Durée des inclusions 18 mois<br>Durée de participation du patient : 3 mois

2. JUSTIFICATION SCIENTIFIQUE ET DESCRIPTION GENERALE DE LA RECHERCHE.

2.1. Contexte de la recherche et revue de la littérature

Les maladies parodontales, gingivites et parodontites, sont des maladies infectieuses d'origine bactérienne à manifestations inflammatoires qui affectent le parodonte. Ces infections sont de type mixte à prédominance anaérobie. Elles se traduisent par des lésions inflammatoires, confinées à la gencive dans le cas des gingivites, ou étendues à l'ensemble des tissus parodontaux dans le cas des parodontites. Elles sont alors notamment responsables d'une perte d'attache parodontale pouvant aboutir à la perte de l'organe dentaire et d'une bactériémie à distance [1].
La prévalence des différentes formes de maladies parodontales de l'adulte de plus de 18 ans varie de 40 à 55% (5 à 10% de parodontites sévères) dans les pays industrialisés [2-4].

L'inflammation et sa résolution apparaissent de plus en plus comme un élément central des maladies parodontales [5-9] et des relations mises en évidence avec de nombreuses pathologies systémiques chroniques [10, 11 , 12, 13, 14 , 15 , 16]. Le traitement parodontal amène à une amélioration significative du taux d'hémoglobine glycquée chez les patients diabétiques [17]. Ainsi, la recherche d'un diabète dans le cadre de la prise en charge d'une maladie parodontale peut être justifiée [18].

2.2. Justification de l'étude

La réaction inflammatoire fait intervenir de nombreux acteurs de la réponse immunitaire, notamment les lymphocytes B (LB). Ce sont les cellules de l'immunité humorale, responsables de la synthèse des immunoglobulines ou anticorps face à la présence d'un antigène. Elles sont caractéristiques d'une inflammation chronique. Il existe plusieurs classes de lymphocytes B caractérisés par des antigènes de surface spécifique (marqueurs membranaires). Une fois activée, ces cellules expriment de nombreux facteurs impliqués, notamment dans l'ostéoclastogènèse (RANKL, TNFα, IL-6, MIP-1α et MCP-3) et participent ainsi aux destructions tissulaires observées dans la parodontite [19, 20].
Lors de la mise en place d'une atteinte parodontale, un important infiltrat inflammatoire est observé au sein de la gencive. Cet infiltrat est caractérisé par la présence de nombreux lymphocytes B issus du sang par diapédèse. Les sous-populations de LB présentes dans le sang et la gencive n'ont été que peu étudiées[21-24]. Cependant, le nombre de LB autoréactifs (CD19+, CD5+) a été rapporté comme étant plus élevé dans le sang des patients souffrant de maladie parodontale [25]. Dans la gencive, le taux des lymphocytes B et T augmente en fonction du niveau de l'inflammation et est corrélé avec la sévérité du processus inflammatoire. L'activation des LB est un préalable à l'évolution de la gingivite vers la parodontite [24]. Ceci suggère qu'il doit exister des lésions stables caractérisées par des LB au repos. Le LB pourrait être alors un indicateur de l'évolution de la maladie, mais aussi permettre d'étudier la réponse au traitement [26]

L'objectif de cette étude pilote est de caractériser les sous-populations des LB présentes chez un patient souffrant d'une parodontite, en fonction du type de maladie parodontale diagnostiqué et de son niveau d'activité, défini sur le plan biologique (MMP8, RANK-L). Nous chercherons ainsi à déterminer si les LB présents ont un phénotype de LB "transitionnel", de LB "immature", de LB "mature" ou de LB "mémoire" à la fois dans le sang

et dans des biopsies gingivales. L'analyse des LB dans le sang pourrait permettre de mettre en évidence l'association d'une sous-population particulière avec l'agressivité de la maladie parodontale et en évidence d'un profil biologique particulier de la réponse de l'hôte, donc du type de parodontite. Nous souhaitons également observer l'évolution de ce phénotype suite à une thérapeutique non chirurgicale classique.
Cette étude permettrait de mieux comprendre la physiopathologie des maladies parodontales et d'affiner le diagnostic, le pronostic et le traitement d'une parodontite, et ainsi participer à la mise en place d'une médecine personnalisée. Un suivi biologique des effets des thérapeutiques pourrait être instauré et permettre de prévenir plus efficacement la récidive.

3. OBJECTIFS

3.1. Objectif principal

L'objectif principal est de déterminer le phénotype et la répartition des lymphocytes B au sein du sang et de la gencive des patients atteints de différentes maladies parodontales.

3.2. Objectifs secondaires

L'objectif secondaire est d'évaluer l'effet du traitement parodontal non chirurgical sur le phénotype et la fonction des lymphocytes B.

4. CONCEPTION ET DEROULEMENT DE LA RECHERCHE

4.1. Méthodologie de la recherche :

Il s'agit d'une étude clinicobiologique prospective multicentrique ouverte, non randomisée, décrivant le phénotype membranaire des lymphocytes B, au niveau du sang et de la gencive, chez des patients atteints de maladie parodontale, et leur évolution avant et après traitement parodontal non chirurgical

4.2. Critères d'Evaluation

Critère principal d'évaluation

Evaluation par cytométrie en flux de l'expression phénotypique des LB du sang périphérique (sang total) et de la gencive.

Critères d'évaluation secondaire

Evaluation par immunofluorescence de la répartition des lymphocytes B et de l'expression des cytokines au sein des tissus gingivaux (analyse en microscopie confocal)
Evaluation par cytométrie en flux du phénotype des lymphocytes T au niveau du sang périphérique
Caractérisation et mesure de différentes cytokines présentes au sein du fluide gingival (technique Luminex®) des patients atteints de maladie parodontale
Analyse microbiologique du biofilm sous-gingival (par PCR quantitaitve) : détermination du pourcentage de parodontopathogènes.

4.3. Déroulement de la recherche

Les patients seront informés de façon complète et loyale, en des termes compréhensibles, des objectifs de l'étude, de leurs droits de refuser de participer à l'étude ou de la possibilité de se rétracter à tout moment. Toutes ces informations figureront sur un formulaire d'information et de consentement remis au patient. Les prélèvements seront conservés au sein de la collection d'échantillons biologiques Humains DC 2014-2158 intitulé « OdontoBrest ».

Inclusion

Après avoir donné son accord, tous les patients suivis à l'hôpital de Brest pourront être inclus dans l'étude. Différents groupes de Parodontite seront comparés à un groupe de témoin (sans parodontite). Un prélèvement sanguin sera réalisé suite à la première consultation et au préalable à la réalisation d'un acte chirurgical afin, entre autres, de déterminer si un diabète n'est pas associé à l'atteinte parodontale, ou s'il n'y a pas de troubles de l'hémostase chez ce patient.

Méthodologie

Le patient est vu en consultation au centre de soins dentaires et une observation clinique de parodontologie est réalisée. Un diagnostic de parodontite peut alors être posée ou non et le patient peut rentrer dans l'étude en tant que ''Parodontite'' ou 'Témoin''. La participation à l'étude ne change pas la prise en charge clinique du patient.

Les prélèvements sont réalisés dans le cadre du parcours de soin du patient dans un but de diagnostic ou de dépistage (diabète et troubles de l'hémostase). Un consentement éclairé informe le patient sur les modalités de prélèvement des échantillons et leur utilisation prévue dans le cadre de la biocollection OdontoBrest (Cf. annexe 6).

L'étude est composée d'une partie observation clinique au centre de soins dentaires de Brest et d'une partie analyses biologiques au laboratoire d'immunologie de Brest.

Les techniques de laboratoire peuvent être réalisés au fur et à mesure de la réception des tubes et à postériori, sur les échantillons congelés dans la biocolection OdontoBrest.

a- En clinique, dans le service d'odontologie

Lors de la prise en charge de patients dans le service d'odontologie, des informations cliniques et biologiques, permettant, en autre, d'établir le diagnostic de maladie parodontale, sont relevés (cf. Annexe : Protocole de prise en charge de patient en parodontologie). Ces informations sont consignées dans le dossier médical du patient sous la forme d'une fiche de recueil des données comprenant les données citées ci-dessous. Les informations relevées durant l'examen clinique sont :

- critères d'inclusion et de non inclusion et questionnaire médical
- les résultats d'analyses biologiques antérieures
- un bilan radiologique de type orthopantomogramme ou bilan long cône, ainsi qu'une analyse écrite

Cette prise en charge suit la méthodologie courante d'évaluation et de diagnostic d'une atteinte parodontale.

Au cours de la prise en charge thérapeutique courante du patient, des prélèvements gingivaux (lors d'extraction dentaire, ou d'assainissement parodontal), bactériens et sanguins seront réalisés.

Le sondage parodontal est réalisé par un seul opérateur, Mademoiselle Camille FRAMERY. L'absence de variations intra-opérateur est vérifiée (calcul Kappa).

a.1 Prélèvement gingival
(cf Annexe 1 : protocole de traitement des échantillons gingivaux)

Le prélèvement gingival est effectué durant une intervention chirurgicale (extraction dentaire, chirurgie parodontale). L'échantillon recueilli correspond à un élément gingival devant être éliminé dans le cadre de l'intervention. Il comprend, si possible, à la fois le tissu conjonctif et les tissus épithéliaux (épithélium gingival et épithélium de poche).
Une fois prélevé, l'échantillon sera placé dans une compresse stérile, imbibée de sérum physiologique et mis dans un tube de transport. Le tube de transport, fermé hermétiquement, sera étiqueté suivant les modalités de la biocollection OdontoBrest et de l'étude (Cf. construction de la numérotation page 13), et sera transmis au laboratoire dans l'heure qui suit le prélèvement

a.2 Prélèvement sanguin
(cf Annexe 2 : protocole de traitement des échantillons sanguins)

Le prélèvement sanguin sera réalisé par l'IDE du service d'odontologie après le diagnostic de maladie parodontale posé ou avant une intervention chirurgicale, chez un patient sain ou un patient atteint de parodontite. Un tube sera transmis au laboratoire d'Immunologie et les autres en hématologie pour une analyse sanguine.

Les prélèvements sanguins sont réalisés dans le cadre de soin.

Le tube pour le laboratoire d'Immunologie sera fermé hermétiquement et étiqueté suivant les modalités de la Biocollection OdontoBrest, et transmis au laboratoire dans l'heure qui suit le prélèvement.

a.3 Prélèvement de fluide gingival
(cf Annexe 3 : protocole de prélèvement de fluide gingival et bactérien)

Un prélèvement de fluide gingival est réalisé lors de l'examen clinique pour évaluer les concentrations de différentes cytokines (IL-1, IL-6, IL-7, IL-8, IL-10, IL-11, IL-12, IL-15, IL-17, sRankl, TNF-α, TGF-β, IFN-γ, MMP8, MMP9, TIMP1, TIMP2). Les prélèvements seront congelés à -80°C. La quantification se fera par la technique multiplex MAP® avec la technologie Luminex® xMAP®. Le volume sera quantifié par pesée des pointes de papiers avant et après prélèvement, avec une balance de précision au 0,001g.

a.4 Prélèvement bactérien
(cf Annexe 3 : Protocole de prélèvement de fluide gingival et bactérien)

Un prélèvement bactérien est réalisé lors de l'examen clinique pour évaluer les bactéries présentes et affiner le diagnostic, et avant une intervention chirurgicale, lors de la réévaluation parodontale. Deux cônes de papiers sont insérés en même temps au niveau du site de prélèvement. Les cônes de papier sont ensuite mis dans des eppendorfs stériles, sans milieu particulier. Les prélèvements sont mis au congélateur à -20°C et seront analysés en PCR quantitative.

Lorsqu'une parodontite est dépistée, le prélèvement se fait au niveau des 3 sites les plus profonds, ou au niveau des dents devant bénéficier d'une intervention chirurgicale (poches profondes).

Une vérification du bon remplissage des documents électroniques, et des fiches de transfert (identification de l'échantillon dans la biocollection et fiche de contrôle et de prévention des incidents) est effectuée avant le transport vers le laboratoire.

b- Au laboratoire d'immunologie de Brest

b.1 Réception des échantillons

Les échantillons sont reçus par le Dr Julien DEMOERSMAN, le Dr Pierre POCHARD ou un personnel du laboratoire d'immunologie. Chaque échantillon est vérifié par le réceptionnaire et tout incident est notifié sur la fiche de signalement d'incident (Cf Annexe 7: Fiche de contrôle et de prévention des incidents)
Les prélèvements gingivaux et bactériens seront stockés à -80°C pour servir à des analyses ultérieures au laboratoire d'immunologie de Brest (type immunofluorescence), ou seront analysés immédiatement (type cytométrie) au laboratoire d'immunologie de Brest.

b.2 Traitement des prélèvements gingivaux
(cf Annexe 1 : Protocole de traitements des prélèvements gingivaux)

Les prélèvements gingivaux sont préparés pour une analyse en cytométrie en flux ou pour une étude en immunofluorescence.

L'analyse en cytométrie est réalisée sur des éluâts de gencive obtenus par broyat. Cette analyse permettra de déterminer et de quantifier les différentes populations et sous-populations de lymphocytes B.

L'analyse en immunofluorescence est réalisée sur des coupes de gencives préparées à partir d'échantillons congelés. Cette analyse par microscope en confocale permettra de déterminer les phénotypes des populations de lymphocytes B et leur répartition au sein des tissus gingivaux. Cette analyse sera réalisée dans les cas où les prélèvements auront été abondants.

b.3 Traitement des prélèvements sanguins
(cf Annexe 2 : Protocole de traitement des échantillons sanguins).

Une fois reçus et vérifiés, les échantillons de sang seront préparés pour les analyses en cytométrie en flux sur sang total. 3 panels de 9 à 10 couleurs sont utilisés pour étudier le phénotype des lymphocytes sanguins.

<u>Suivi</u>

Le suivi de la parodontite ne sera pas modifié par le protocole, la réévaluation aura lieu à 3 mois en consultation de routine pour évaluer le résultat du traitement parodontal non chirurgical. Lors de cette consultation, où une nouvelle observation clinique sera réalisée, de nouveaux prélèvements sanguins et gingivaux pourront êtres obtenus si une intervention de chirurgie buccale était indiquée.

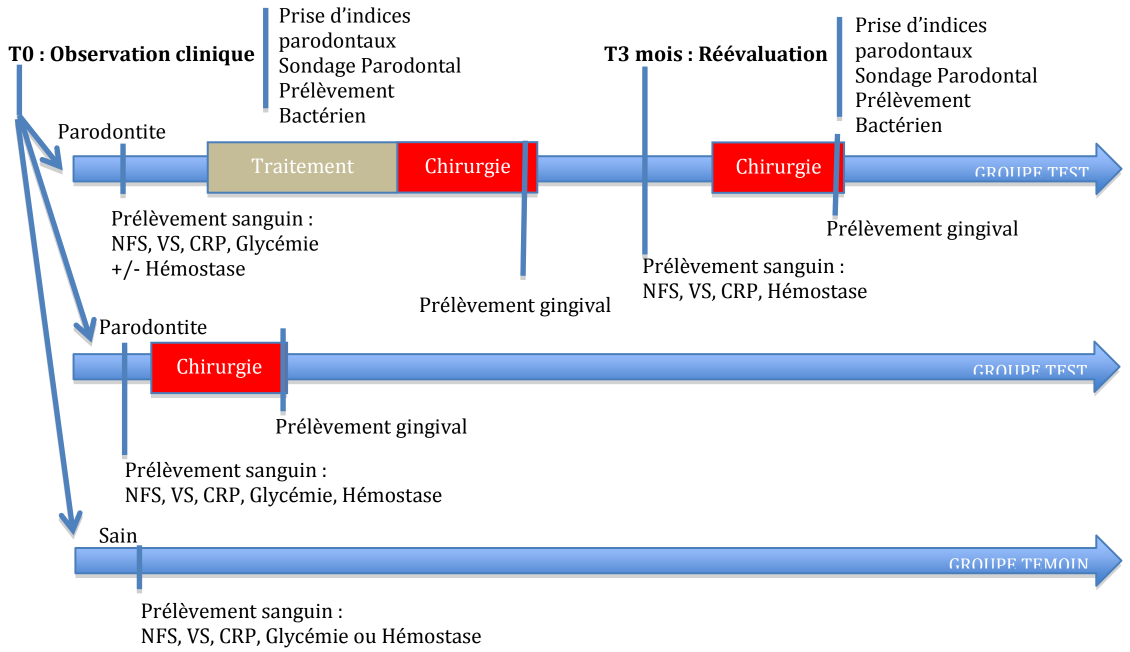

Temps possibles d'observation clinique et prélèvements durant la prise en charge d'un patient

Le sang restant dans le tube sera centrifugé afin de séparer les cellules du plasma qui seront congelées ensuite dans le cadre de la biocollection OdontoBrest. Les cellules et le plasma congelés serviront pour des analyses ultérieures (PCR Quantitative et analyses des protéines circulantes).

5. POPULATION ETUDIEE

5.1. Description de la population source

La population source est constituée de sujets venant consulter dans le service d'odontologie de Brest.

Cas : Patients souffrants de maladie parodontale chronique ou agressive incluant la réalisation d'une analyse sanguine.

Témoins : Patients ne souffrant pas de parodontite mais devant subir une extraction dentaire ou une chirurgie mucogingivale incluant la réalisation d'une analyse sanguine.

5.2 Critères d'inclusion et de non inclusion

Inclusion

- Cas :
  - âgé de plus de 18 à 75 ans
  - souffrant d'une parodontite chronique ou agressive
  - nécessitant une chirurgie type parodontale
  - avec une suspicion de troubles de l'hémostase ou de la présence d'un diabète
  - en bonne santé général
  - ayant donné son consentement

- Témoin :
  - majeur
  - Nécessitant une extraction dentaire ou une chirurgie mucogingivale
  - avec une suspicion de troubles de l'hémostase ou de la présence d'un diabète
  - en bonne santé général
  - ayant donné son consentement

Non-inclusion-Exclusion

- Cas :
  - mineur
  - prise d'antibiotiques dans les 3 mois qui précèdent l'étude
  - femme enceinte
  - pathologie systémique, notamment une pathologie inflammatoire chronique
  - en cours de traitement orthodontique
- Témoins:
  - mineur
  - souffrant de parodontite
  - prise d'antibiotiques dans les 3 mois qui précèdent l'étude
  - femme enceinte
  - pathologie systémique, notamment une pathologie inflammatoire chronique
  - en cours de traitement orthodontique

Le tabagisme sera pris en compte dans les différents groupes.

5.3 Faisabilité

Les maladies parodontales ne sont pas rares et leur prise en charge est fréquente au centre de soins dentaires.

Une analyse intermédiaire sera réalisée afin d'évaluer en cours d'étude s'il est nécessaire d'inclure un plus grand nombre de sujets.

6. MODALITES DE RECRUTEMENT ET D'INFORMATION DES PERSONNES CONCERNEES

Un patient venant consulter au centre de soins et chez qui une parodontite, ou non, est diagnostiquée et nécessitant un prélèvement sanguin en vue de s'assurer de l'absence d'un diabète ou de troubles de l'hémostase pourra être recruté pour l'étude. Une fois les critères de sélection vérifiés, le protocole lui sera exposé et son accord de participation à l'étude sera recherché pour son inclusion (consentement signé). Le consentement du patient sera ainsi recherché dès le début de sa prise en charge.
Le protocole ne modifiant pas la prise en charge du sujet, il n'y a aucune pénibilité particulière pour le patient, ni aucune perte de chance possible

7. STATISTIQUES

7.1. Responsable de l'Analyse et lieu de conservation des données

Pr Jacques Olivier PERS, CHRU de Brest, Hôpital Morvan – Laboratoire d'Immunologie et Centre de Soins Dentaires – 2 av Foch – 29609 Brest Cedex

7.2. Justification du nombre de sujets à inclure

Cette étude concerne 60 sujets, patients et témoins. En effet, 15 sujets par groupe (parodontite agressive, parodontite chronique) et 30 patients sans parodontite appariés en âge et en sexe seront suffisants pour répondre aux objectifs fixés.

La limite de 15 patients par groupe de patients souffrant de parodontite se justifie par un nombre moins important de patients souffrant de parodontite agressive.

7.3. Méthode d'analyse statistique

Comparaison de la fréquence des différentes sous-populations de LB sanguins, définie par l'expression de marqueurs membranaires, avant et après thérapeutique initiale. Les témoins sont des patients sans parodontite et sans pathologie systémique.
Une analyse intermédiaire sera réalisée afin d'évaluer en cours d'étude s'il est nécessaire d'inclure un plus grand nombre de sujets.

Méthode d'analyse
Les variables qualitatives seront décrites en terme d'effectif, moyenne et écart type. Les variables qualitatives seront décrites en terme d'effectif et de proportion.

Les variables quantitatives paramétriques seront comparées entre les groupes par des tests de students non appariés et les variables non paramétriques par des tests de Mann-Withney. Les variables qualitatives seront comparées par des tests de khi-2 ou des tests de Fisher, en cas de petits effectifs.

Dans le cas de plusieurs groupes à comparer (patients avec parodontite agressive, patient avec parodontite chronique, patients sans parodontite), un ANOVA sera réalisé ou un test de Kruskal Wallis dans les cas de donnés non paramétriques.

La limite de signification statistique est fixée à 0,005 pour l'ensemble des tests réalisés. Les analyses statistiques seront réalisées grâce au logiciel graphpad IBM SPSS au sein de l'EA2216 INSERM, ERI29, sous la responsabilité de Jacques-Olivier PERS.

8. TRAITEMENT DES DONNEES ET CONSERVATION DES DOCUMENTS ET DES DONNEES RELATIVES A LA RECHERCHE

8.1 Cahiers d'observation (CRF) (Cf Annexe 4 : Protocole de gestion de données patients)

Les données patients et les données de l'étude seront consignées électroniquement dans une base de données informatique au format Excel. En parallèle, deux classeurs sont mis à disposition des différents protagonistes :

- un classeur au centre de soins dentaires de Brest contenant :
  - des fiches vierges de consentement des patients de la biocollection
  - une copie des fiches de consentement signées par les patients (l'original sera conservé dans le dossier clinique du patient au CSD)
  - fiche de recueil des données comprenant les critères de sélection et questionnaire médical
  - une table de correspondance : numéro de biocollection, identité du sujet et numéro de dossier médical.
  - des fiches vierges de contrôle et de prévention des incidents
  - les protocoles de prise en charge de patients au CSD dans le cadre de l'étude
  - les protocoles de traitement des échantillons
  - les protocoles de gestion des données de l'étude
- un classeur au laboratoire d'immunologie de Brest contenant :
  - les fiches de contrôle et de prévention des incidents remplis par échantillons reçus
  - les protocoles de prise en charge de patients au CSD dans le cadre de l'étude
  - les protocoles de traitement des échantillons
  - les protocoles de gestion des données de l'étude

Un fichier électronique en format Excel sera ouvert pour l'étude (dossier informatique). Toutes les informations requises par le protocole y seront inscrites.(dossier protocoles). Il reprendra les différentes données cliniques et biologiques du patient décrites dans le protocole. Il doit comprendre les données nécessaires pour confirmer le respect du protocole, déceler les écarts majeurs au protocole et toutes les données nécessaires aux analyses. Le fichier électronique sera en partage avec le laboratoire d'immunologie et dans le centre participant à l'étude.

Il comprendra un fichier Excel anonymisé (OdontoBrest – Patients.xlsx) relié à la biocollection, un dossier comprenant les documents et les protocoles de l'étude LBParo et de

la biocollection OdontoBrest et deux dossiers d'enregistrement des données cliniques nommés :

- patients étude LBParo–ID clinique, comprenant un sous dossier par patient avec, entre autres, le fichier excel ChartingParo.xlxs du patient
- patients étude LBParo –ID biocollection, comprenant les mêmes données que le précédent dossier mais sans possibilité d'identifier les patients

Le numéro de "biocollection- étude " sera inscrit sur le consentement et inclus dans le dossier médical du patient. Ainsi lorsque le patient décide de se retirer , le clinicien communiquera le numéro de "biocollection- étude " et les données seront supprimées de la base.

Le dossier LBParo- ID biocollection sera un dossier partagé en lecture seule avec le laboratoire d'immunologie de Brest, et au niveau d'un ordinateur dédié.

La personne responsable du remplissage des CRF électronique et des documents papiers de la biocollection sera Mlle Camille FRAMERY, étudiante en chirurgie dentaire. Les données manquantes seront notifiées dans la fiche de contrôle et de prévention des incidents.

Cheminement des données cliniques entre le centre de soins dentaires et le laboratoire d'immunologie

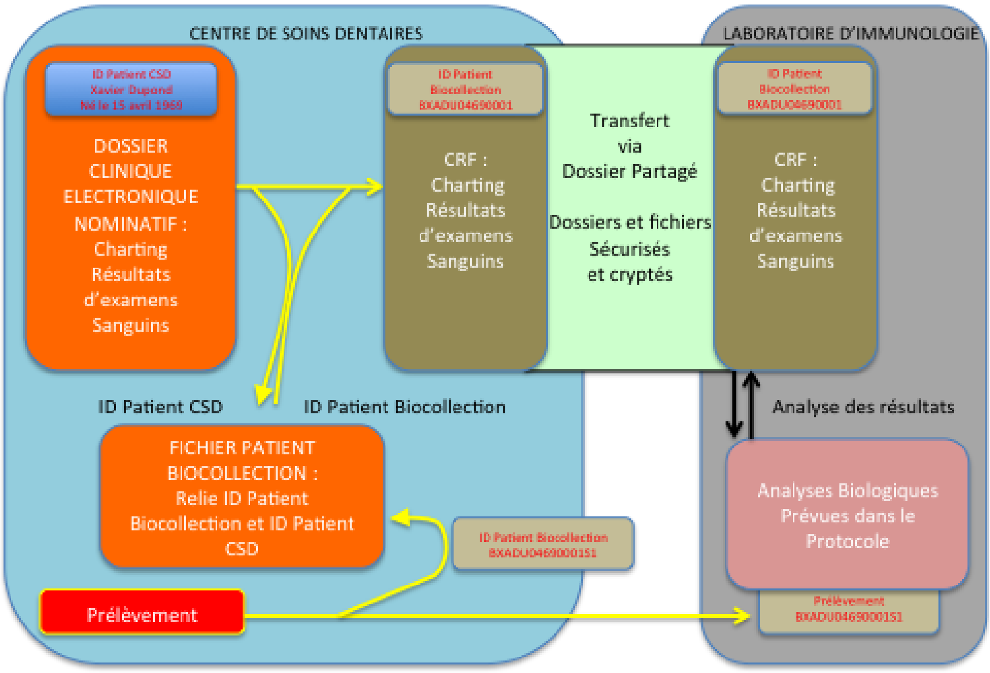

Construction du numéro de la biocollection : ex Xavier Dupond, né le 15 avril 1969

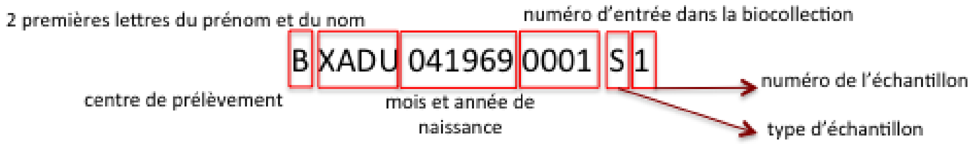

8.2. Identification des données recueillies dans le cahier d'observation

Les données recueillies dans le cahier d'observation seront des :
Données cliniques :
- antécédents médicaux
- habitudes de vie : tabagisme
- charting parodontal, indices parodontaux
- diagnostic / pronostic

Données biologiques :
- résultats des bilans sanguins

Données liées à l'étude :
- poids et site des prélèvements de fluide gingival
- sites des prélèvements bactériens
- sites des prélèvements gingivaux

8.2 Confidentialité

Les données sources sont conservées au sein des centres des soins participants à l'étude, dans le CRF électronique, et les dossiers cliniques des patients.

Les personnes ayant un accès direct prendront toutes les précautions nécessaires en vue d'assurer la confidentialité des informations relatives aux personnes qui s'y prêtent et notamment en ce qui concerne leur identité ainsi qu'aux résultats obtenus.
Ces personnes, au même titre que les investigateurs eux-mêmes, sont soumises au secret professionnel (selon les conditions définies par les articles 226-13 et 226-14 du code pénal).
Pendant la recherche ou à son issue, les données recueillies sur les personnes qui s'y prêtent et transmises par les intervenants seront rendues codées.

Afin de maintenir une confidentialité maximale, un numéro unique sera attribué au patient. Il se construira de la manière suivante, sur l'exemple de B XADU 041969 0001 S 1, où :
- B correspond au centre participant à l'étude, si à terme, plusieurs centres alimentaient la biocolection :
    - B pour Brest
- XADU correspond au deux premières lettres du nom et du prénom du patient
- 041969 correspond à la date de naissance du patient
- 0001 correspond au numéro d'entrée du patient dans la biocollection
- S correspond à la nature de l'échantillon :
    - S pour sang
    - G pour la gencive
    - B pour les bactéries
    - F pour le fluide gingival
- 1 correspond au numéro de l'échantillon

8.3. Déclaration CNIL

Une déclaration normale relative aux droits des personnes participant à cette recherche sont intégrées dans la non-opposition sera réalisée auprès de la Commission Nationale Informatique et Libertés (CNIL) relative au traitement des données à caractère personnel ayant pour finalité la recherche.

Les informations relatives aux droits des personnes participant à cette recherche sont intégrées dans la non-opposition.

8.4. Traitement des données

La collecte des données cliniques reposera sur la mise en place d'une base de données clinique informatique avec la création de masques de saisie à l'image du cahier d'observation en conformité avec le protocole et les réglementations actuellement en vigueur.

Un contrôle qualité final des données nécessaires à l'analyse statistique sera mis en place selon la procédure actuellement en vigueur.

La saisie des données cliniques sera effectuée par Mlle FRAMERY et la saisie des données biologiques par le Dr DEMOERSMAN. Les techniques de biologie seront réalisées par le Dr DEMOERSMAN, le Dr POCHARD ou un membre du laboratoire d'immunologie.

L'analyse des données sera réalisée par le Pr PERS, le Dr. DEMOERSMAN, le Dr POCHARD et Mlle FRAMERY.

8.4. Archivage des données

Tous les cahiers et documents de l'étude devront être conservés dans une armoire fermée à clé, et archivés par l'investigateur jusqu'à publication des résultats ou durant 5 ans après la fin de la recherche.

9. FINANCEMENT

9.1. Ressources financières

L'ensemble du protocole sera financé par l'EA2216, INSERM ESPRI ERI29.

l'EA2216, INSERM ESPRI ERI29 établira des contrats de prestation de service pour les analyses de bactériennes avec Rennes et de fluide gingival avec Nantes, dans le cadre de leurs coopérations déjà existantes (y compris les frais de transport).

9.2. Incidence financière

→ pour l'établissement

→ pour la CPAM

10. PUBLICATION

Les communications et publications seront réalisées sous la responsabilité du coordonnateur de l'étude avec l'accord des investigateurs associés. Les coauteurs des publications seront les investigateurs impliqués. Les termes de « CHU de Brest » devront apparaître dans l'adresse des auteurs. Une copie des publications devra être adressée à la Délégation à la Recherche clinique.

11. BIBLIOGRAPHIE

1. Pihlstrom, B.L., B.S. Michalowicz, and N.W. Johnson, *Periodontal diseases.* Lancet, 2005. **366**(9499): p. 1809-20.
2. Albandar, J.M., *Periodontal diseases in North America.* Periodontol 2000, 2002. **29**: p. 31-69.
3. Hugoson, A. and O. Norderyd, *Has the prevalence of periodontitis changed during the last 30 years?* J Clin Periodontol, 2008. **35**(8 Suppl): p. 338-45.
4. Bourgeois, D., P. Bouchard, and C. Mattout, *Epidemiology of periodontal status in dentate adults in France, 2002-2003.* J Periodontal Res, 2007. **42**(3): p. 219-27.
5. Kantarci, A. and T.E. Van Dyke, *Resolution of inflammation in periodontitis.* J Periodontol, 2005. **76**(11 Suppl): p. 2168-74.
6. Van Dyke, T.E., *Control of inflammation and periodontitis.* Periodontol 2000, 2007. **45**: p. 158-66.
7. Van Dyke, T.E., *The etiology and pathogenesis of periodontitis revisited.* J Appl Oral Sci, 2009. **17**(1).
8. Van Dyke, T.E., *Proresolving lipid mediators: potential for prevention and treatment of periodontitis.* J Clin Periodontol, 2011. **38 Suppl 11**: p. 119-25.
9. Stashenko, P., et al., *Inflammation and genetic risk indicators for early periodontitis in adults.* J Periodontol, 2011. **82**(4): p. 588-96.
10. Almas, K., et al., *The relationship between periodontal disease and blood glucose level among type II diabetic patients.* J Contemp Dent Pract, 2001. **2**(4): p. 18-25.
11. Klebanoff, M. and K. Searle, *The role of inflammation in preterm birth--focus on periodontitis.* BJOG, 2006. **113 Suppl 3**: p. 43-5.
12. Hingorani, A.D. and F. D'Aiuto, *Chronic inflammation, periodontitis and cardiovascular diseases.* Oral Dis, 2008. **14**(2): p. 102-4.
13. Van Dyke, T.E., *Resolution of inflammation-unraveling mechanistic links between periodontitis and cardiovascular disease.* J Dent, 2009. **37**(8): p. S582-3.
14. Goldie, M.P., *The relationship of inflammation to systemic diseases and chronic periodontitis.* Int J Dent Hyg, 2010. **8**(3): p. 253-5.
15. Rethman, M.P., *Inflammation in chronic periodontitis and significant systemic diseases.* J Calif Dent Assoc, 2010. **38**(4): p. 247-57.
16. Taylor, B., et al., *The effect of initial treatment of periodontitis on systemic markers of inflammation and cardiovascular risk: a randomized controlled trial.* Eur J Oral Sci, 2010. **118**(4): p. 350-6.
17. Simpson, T.C., et al., *Treatment of periodontal disease for glycaemic control in people with diabetes.* Cochrane Database Syst Rev, 2010(5): p. CD004714.
18. Katagiri, S., et al., *Improvement of glycemic control after periodontal treatment by resolving gingival inflammation in type 2 diabetic patients with periodontal disease.* J Diabetes Investig, 2012. **3**(4): p. 402-9.
19. Han, X., et al., *Bacterial-responsive B lymphocytes induce periodontal bone resorption.* J Immunol, 2006. **176**(1): p. 625-31.
20. Kawai, T., et al., *B and T lymphocytes are the primary sources of RANKL in the bone resorptive lesion of periodontal disease.* Am J Pathol, 2006. **169**(3): p. 987-98.
21. Seymour, G.J. and J.S. Greenspan, *The phenotypic characterization of lymphocyte subpopulations in established human periodontal disease.* J Periodontal Res, 1979. **14**(1): p. 39-46.
22. Cindric, G., et al., *Lymphocyte subpopulations and the expression of intercellular adhesion molecules in chronic periodontitis.* Coll Antropol, 2004. **28**(2): p. 825-32.

23. Sigusch, B.W., et al., *Evidence for a specific crevicular lymphocyte profile in aggressive periodontitis.* J Periodontal Res, 2006. **41**(5): p. 391-6.
24. Donati, M., et al., *B-1a cells and plasma cells in periodontitis lesions.* J Periodontal Res, 2009. **44**(5): p. 683-8.
25. Berglundh, T., et al., *The presence of local and circulating autoreactive B cells in patients with advanced periodontitis.* J Clin Periodontol, 2002. **29**(4): p. 281-6.
26. Suzuki, J.B., et al., *Effect of periodontal therapy on spontaneous lymphocyte response and neutrophil chemotaxis in localized and generalized juvenile periodontitis patients.* J Clin Periodontol, 1985. **12**(2): p. 124-34.

12. ANNEXES

Annexe 1
Protocole de traitement des échantillons gingivaux

En clinique :

- Explication de l'étude, de son intérêt et de ses objectifs
- Signature du consentement (Consentement Biocollection OdontoBrest)
- Réalisation de l'intervention chirurgicale prévue
- Prélèvement d'un fragment de gencive de 5*5 mm minimum contenant à la fois de l'épithélium et le tissu conjonctif, dans le cadre de l'intervention
- Placement de l'échantillon dans une compresse stérile imbibée de sérum physiologique et placement dans un sachet de transport.
- Étiquetage du sachet : numéro de l'échantillon dans la biocollection – date de prélèvement -
- Remplissage du fichier excel ''OdontoBrest-Patients.xlsx'' : identité du patient et numéro des échantillons lui correspondant. Ce fichier ne sera accessible que par les investigateurs du centre de soins dentaires
- Remplissage de la fiche de contrôle et de prévention des incidents
- Appel du laboratoire d'immunologie pour prévenir de l'arrivée de l'échantillon
- Transport de l'échantillon et de la fiche de contrôle et de prévention des incidents au laboratoire

Au laboratoire :

- Réception de l'échantillon
- Vérification et remplissage de la fiche de contrôle de prévention des incidents
- Rangement de la fiche de contrôle de prévention des incidents dans le classeur approprié Biocollection OdontoBrest

Matériel :
- Classeur biocollection OdontoBrest

Préparation de l'échantillon

L'échantillon est utilisé de suite pour une analyse en cytométrie en flux pour les échantillons issus du CSD de Brest.

Les échantillons issus de Nantes ou certains échantillons de Brest sont préparés pour l'analyse en immunofluorescence

<u>Préparation de l'échantillon pour une analyse en cytométrie en flux : élution de gencive</u>

La biopsie est conservée à 4°C dans du Sérum de veau fœtal (SVF) décomplémenté. Le SVF décomplémenté et le RPMI 1640 doivent être préparés à l'avance.

1- Pesée de l'échantillon (balance de précision 0,0001g)
2- Laver l'échantillon gingival avec du Hank's
3- Couper la biopsie dans une boîte de pétri en très petits bouts à l'aide d'un scalpel stérile.
4- Broyer la gencive dans 2 ml de RPMI 1640 (doit être à température ambiante) avec un Dounce glass homogenizer ensuite directement avec le filtre dans une boite de pétri.
5- Filtrer ces 2 ml avec un filtre de 70 µm et rincer avec du Hank's, le dounce et le passer dans le filtre.
6- Récupérer l'ensemble du liquide contenu dans la boite de pétri
7- Laver les cellules avec du Hank's (doit être froid) par centrifugation 10 min à 1800 rpm à 4°C – programme 14
8- Eliminer le surnageant par retournement et laver une 2e fois.
9- Eliminer le surnageant par retournement
10- Resuspendre les cellules dans 1 ml de Hanks ou PBS (doit être froid) et les mettre dans la glace
11- Faire une numération cellulaire (20 µl de suspension cellulaire + 20 µl de bleu trypan + 160 µl de PBS)
12- Reprendre les cellules dans un tube de cytométrie en utilisant un filtre fin (External Filter Tips, Laboscience INC, OFT200245/MHC) à ici 500 µl par tube
13- Lavage des cellules à 1500 rpm 5min à 4°C – prog 19 –
14- Retirer le surnageant et les reprendre dans 100 µl
15- Ajouter les anticorps de marquage (voir tableau suivant pour le panel)
16- Passer les tubes dans le cytomètre et utilisation du programme LBPARO-gencive.pro

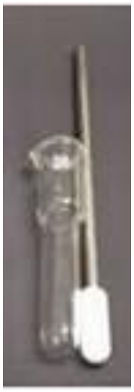

Matériel :
- Hank's
- RPMI1640
- SVF Décomplémenté
- Boite de pétri
- Précelle
- Scalpel stérile
- Filtre BD Falcn Cell Steiner
- Filtre external filter tips
- Dounce Glass homogenizer
- Tubes de cytométrie
- Eppendorfs
- Falcons

17- Sauvegarde des LMD dans le répertoire correspondant à l'étude sur l'ordinateur du laboratoire. Les fichiers enregistrés doivent comprendre : numéro de biocollection et date de prélèvement

<u>Préparation de l'échantillon pour une analyse en immunofluorescence :</u>

<u>Congélation du fragment</u>

<u>Protocole</u>

Il est préférable de travailler sous hotte chimique

Une fois la biopsie prélevée :

Remplir le récipient d'azote liquide

Remplir le bécher d'iso-pentane et mettre ce dernier à refroidir au contact avec l'azote liquide (au minimum 1/3 du bécher doit être immergé dans l'azote liquide).

L'iso-pentane est à la température idéale pour la congélation, lorsqu'il commence à se solidifier (présence de cristaux blancs au fond du bécher).

Mettre une grosse goutte de résine de scellement dans une nacelle de pesée et déposer la biopsie à l'intérieur de la goutte de résine. Attention,
éviter les bulles dans la résine.

Plonger la nacelle dans l'iso-pentane refroidi pendant 2 à 3 minutes.

Sortir la nacelle de l'iso-pentane et rogner au scalpel la résine autour de la biopsie afin qu'elle rentre dans le cryotube.

Conserver immédiatement la biopsie à –80°C.

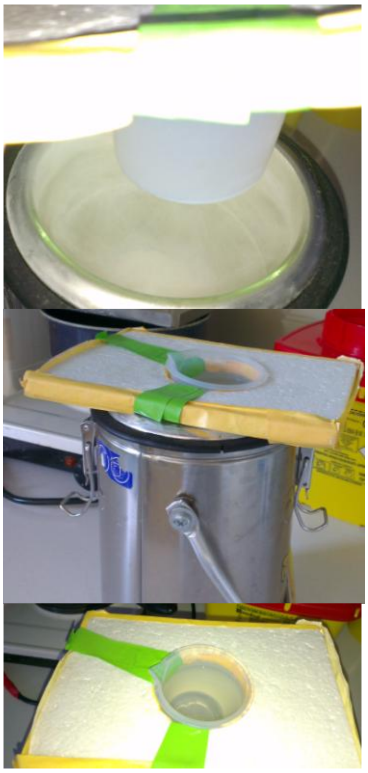

<u>Matériel :</u>

- un bécher de 100 ml en plastique épais
- une plaque de polystyrène présentant un trou permettant de soutenir le bécher en plastique au-dessus d'un récipient contenant de l'azote liquide (petite boîte en polystyrène ou thermos adapté)
- iso-pentane (2-méthyl-butane GPR RECTAPUR®, VWR référence 24872-298)
- azote liquide
- résine de scellement (Shandon Cryomatrix, ThermoScientific référence 6769003).
- scalpels
- cryotubes préalablement étiquetés avec l'identifiant du patient dans la biocollection et indiquant le tissu prélevé

<u>Transport</u>

Au moment de l'envoi, mettre le cryotube contenant le prélèvement dans un petit sac en plastique contenant de la carboglace.

Enfouir le tout sous une quantité suffisante de carboglace dans une boîte en polystyrène expansé.

Sceller la boîte avec de l'adhésif.

Attention, utiliser un contenant qui respire (polystyrène) et n'est pas totalement hermétique, sinon risque d'explosion.

Avertir le laboratoire receveur, envoyer le prélèvement en début de semaine de façon à ce qu'il soit réceptionné et transféré dans un congélateur ou une cuve à azote liquide dès son arrivée.

Envoyer par transport express type chronopost avec livraison en mains propres.

<u>Cryosection des fragments congelés</u>

Utiliser la machine de coupe pour réaliser de coupes fines de

<u>Colorations des cryosections</u>

Le jour d'avant

Préparer PFA 4% dans du PBS et le filtrer. Le conserver dans le noir à 4°C. (200 ml sont nécessaires au minimum pour réaliser un bain de fixation).

Note : il vaut mieux utiliser du PFA frais (il doit être préparé tous les 3 jours)

La formaldéhyde est toxique. Utiliser uniquement une hotte aspirante pour préparer PFA

1- Préparer du PBS frais : 1l d'eau + poudre pour PBS
2- Prendre la poudre pour PFA dans la chambre froide

Sous la hotte, pour réaliser 100 ml de PFA à 4%

1- Faire chauffer la casserole avec de l'eau pour faire chauffer à bain-marie
2- Peser 4 g de poudre
3- Dans un bécher, mettre la poudre et 100 ml de PBS, bien mélanger
4- Mettre à chauffer environ 30 minutes jusqu'à obtenir un mélange translucide
5- Filtrer le mélange dans le flacon en verre
6- Fermer le flacon, le nommer et le dater
7- Laisser refroidir au réfrigérateur jusqu'à utilisation

Matériel :
PBS frais
Poudre pour PFA
Becher
Casserole
Flacon Verre + Bouchon hermétique
Pipette Man + pipette de 50 ml
Entonnoir
Cône papier de filtration

Le jour J

Ne jamais se retrouver avec un dessèchement de l'échantillon

Salle avec hotte aspirante, sous la hotte

1- Mettre les lames le bac en verre
2- Rincer brièvement et avec attention les tissus dans du PBS (Il doit être préparé le jour J)
3- Fixer avec du PFA 4% la cryosection (en bain ou directement dans la glissière) pendant 15 minutes à température ambiante
4- Rincer brièvement avec du PBS les lames 2 fois, pour éliminer l'excès de PFA (mettre le PFA+PBS dans le bidon de recyclage, sous la hotte)
5- Conserver les lames dans le bac dans du PBS pour le transport dans la salle d'immunohistochimie

Matériel :
PBS frais
PFA 4% frais
Anticorps primaire
Anticorps secondaire
Micropipettes
Pipette Man
Sopalin
Bac en verre pour mettre les lames
Plat en métal
Eppendorfs
Crayon gris
Super Pat Pen

Salle immunohistochimie

6- Rincer 3 fois dans du PBS pendant 5 minutes à chaque fois sur les plateaux elliptiques
7- Pendant les phases de rinçage, préparer les anticorps pour la coloration : dilution en fonction de l'anticorps dans 100 µl (cd20= 1/10), dans un tube eppendorf
8- Essuyer la plaque avec du sopalin, sans toucher l'échantillon
9- Poser les échantillons sur le plateau en métal avec 2 pipettes+ scotch + sopalin exprimé de PBS (atmosphère humide). Couvrir d'un film aluminium
10-Délimiter avec le Super Pat Pen une zone autour de l'échantillon
11- Incuber avec le premier anticorps primaire (dilué dans du PBS) pendant 40 minutes à température ambiante. Poser une petite quantité sur l'échantillon.
12- Rincer 3 fois dans du PBS pendant 5 minutes à chaque fois
13- Incuber avec le second anticorps primaire (dilué dans du PBS) pendant 40 minutes à température ambiante
14- Rincer 3 fois dans du PBS pendant 5 minutes à chaque fois
15- Incuber l'anticorps secondaire conjugué au fluorochrome (dilué dans le PBS) pendant 40 minutes à température ambiante
16- Rincer 3 fois dans du PBS pendant 5 minutes à chaque fois
17- Monter la lamelle avec une goutte de montage - glycérine tamponnée - (Vectashield)

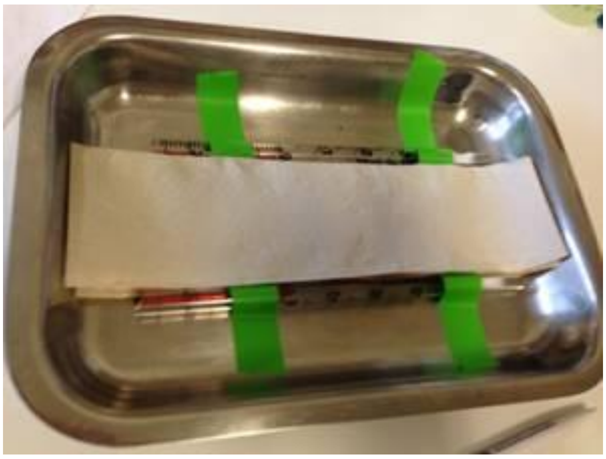

Panel d’analyse des échantillons gingivaux – Cytométrie en flux -

			Laser 1 488 nm				Laser 2 638 nm		Laser 3 405 nm		Laser 1	Laser 2	Protocole Cytométrie
**Fluorochrome**			**PE**	**FITC**	**PC5,5**	**PC7**	**APC**	**AAF750**	**PB**	**KO**	**ECD**	**AAF700**	
Volume d'Eluat (microL)			Gencive										
**Panel G**		tube Rankl	Rankl	CD45	CD27	CD38	IgD	DRAQ7	IgM	CD8	CD5	CD19	**LBParo-gencive.pro**
vol (microL)	100		20	5	3,5	3,5	3,5	3	10	5	10	3,5	
		tube isoRankl	iso	CD45	CD27	CD38	IgD	DRAQ7	IgM	CD8	CD5	CD19	**LBParo-gencive.pro**
vol (microL)	100		5	5	3,5	3,5	3,5	3	10	5	10	3,5	
Origine			PE254/ PE25	FITC 45	PC5, 5 27	PC7 38	APC A5	NC	PB B3	KO 8	ECD 5	AAF700 19	

Panel d’analyse des échantillons gingivaux – Immunofluorescence -

Mouse anti human **CD5**	*65*	1/25e	Donkey anti-mouse IgG (H+L) **FITC**	*1/200e*	361
Mouse anti human **CD19**	*202*	1/100e	Donkey anti-mouse IgG (H+L) **Rhodamine**	*1/200e*	299

Annexe 2
Protocole de traitement des échantillons sanguins

En clinique :

- Explication de l'étude, de son intérêt et de ses objectifs
- Signatures du consentement (Consentement Biocollection OdontoBrest et Consentement de l'étude LBParo)
- Prescription de la prise de sang : NFS, VS, CRP, Plaquettes, Cholestérol total-HDL, Triglycérides, Glycémie à jeun et/ou Bilan de l'Hémostase (TCA, TP)
- Réalisation de la prise de sang par une IDE du CSD – 1 tube est prélevé pour l'étude (tube mauve) –
- Étiquetage du tube : numéro de l'échantillon dans la biocollection – date de prélèvement
- Remplissage du fichier excel ''OdontoBrest-Patients'' : identité du patient et numéro des échantillons lui correspondant. Ce fichier ne sera accessible que par les investigateurs du centre de soins dentaires
- Remplissage de la fiche de contrôle et de prévention des incidents
- Appel du laboratoire d'immunologie pour prévenir de l'arrivée de l'échantillon
- Transport de l'échantillon et de la fiche de contrôle et de prévention des incidents au laboratoire

Au laboratoire :

Matériel :

- Classeur biocollection OdontoBrest

- Réception de l'échantillon
- Vérification et remplissage de la fiche de contrôle de prévention des incidents
- Rangement de la fiche de contrôle de prévention des incidents dans le classeur approprié

1. Préparation de l'échantillon pour l'analyse en cytométrie en flux

Matériel :

- Tubes de cytométrie : 1 par groupe de marquage, 3 panels (6 tubes par patients)
- PBS ou Hank's

1. Prendre 100 µl de sang et les diluer dans 4 ml de PBS ou de Hank's
2. Centrifuger à 200g (1200 rpm), pendant 5 minutes
3. Retirer le surnageant de Hank's ou PBS
4. Recommencer l'opération 2 fois
5. Ajouter les anticorps de marquage (voir tableau suivant, en fonction du panel)
6. Placer les tubes pour la lyse des cellules (environs 1min/tube) dans la machine de lyse (TQ-Prep)
7. Passer les tubes dans le cytomètre et utilisation des programmes suivants :
   - panel B : LBPARO-panelB.pro
   - panel B : LBPARO-panelB1.pro
   - panel T : LBPARO-panelT.pro
8. Sauvegarde des LMD dans le répertoire correspondant à l'étude sur l'ordinateur du laboratoire. Les fichiers enregistrés doivent comprendre : numéro de biocollection et date de prélèvement

2. Préparation de l'échantillon pour la conservation du sérum et la conservation des cellules sanguines dans le cadre de la biocollection OdontoBrest

A. Récupération du sérum (P)

1. Centrifuger les tubes de sang 10 min à 3000 rpm à température ambiante
2. Décanter le plasma et le placer dans un falcon de 10 ml
3. Étiqueter le falcon avec le numéro de la biocollection OdontoBrest et la date de congélation
4. Placer l'échantillon dans le -80 °C
5. Remplir la fiche de recensement des échantillons de la biocollection et la placer dans le classeur Biocollection OdontoBrest
6. Remplir le fichier Excel OdontoBrest-LBParo.xlsx pour le recensement des échantillons de la biocollection
7. Signaler éventuellement un incident survenu durant la préparation sur la fiche de contrôle et de prévention des incidents correspondant à la biocollection

B. Récupération des cellules sanguine (CS) – Après récupération sérum -

1. Préparer milieu de congélation 2X constitué de 20% de DMSO dans du sérum bovin fœtal (FBS). Ce milieu peut être stocké pendant 1 semaine à 4 ° C
2. Décanter le plasma et le placer dans un falcon de 10 ml
3. Préparer "Mr.Frosty": Retirez le support en polyéthylène haute densité flacon et l'insert en mousse de l'unité de polycarbonate. Ajouter 250 ml de 2-propanol à 100% jusqu'à la limite de remplissage (NE PAS REMPLIR). Évitez d'asperger les étiquettes le 2-propanol car l'alcool peut dissoudre d'encre. Remplacer l'alcool au bout de 5 utilisations et documenter un changement de réactif.
4. Centrifuger les cellules à 300 g pendant 10 min à température ambiante
5. Resuspendre les cellules suivant une concentration de $2x10^7$ cellules / ml dans un milieu de culture cellulaire à la température ambiante. Si le nombre de cellules est inférieur à $1x10^7$, resuspendre les cellules dans 0,5 ml.
6. Ajouter au goutte à goutte suffisamment de milieu de congélation 2X à température ambiante pour doubler le volume de la suspension cellulaire. Remuer doucement le tube lors de l'ajout du milieu de congélation.
7. Retirer lentement la suspension cellulaire à l'aide d'une pipette et distribuer 1 ml par tube cryogénique.
8. Placer immédiatement les cryotubes dans un conteneur avec gel Mr. Frosty à température ambiante et placez le récipient de congélation dès que possible dans le congélateur à -80 ° C.

9- Transférer les tubes cryogéniques dans un réservoir d'azote liquide après 1 à 14 jours de congélation.

Matériel :

- Dimethyl sulfoxide, DMSO (Sigma-Aldrich, D8418)
- Inactivated fetal bovine serum (FBS)
- Culture media supplemented with glutamine, inactivated FBS and antibiotics (RPMI-1640 or DMEM)
- Cryopreservation vials (Corning life science, product #430488)
- 2-propanol (Sigma-Aldrich, I9516)
- Mr Frosty™ freezing container (Thermo-scientific, 5100-0001)

Panels d'analyse des échantillons sanguins

			Laser 1 488 nm				Laser 2 638 nm		Laser 3 405 nm		Laser 1	Laser 2	Protocole Cytométrie
**Fluorochrome**			**PE**	**FITC**	**PC5,5**	**PC7**	**APC**	**AAF750**	**PB**	**KO**	**ECD**	**AAF700**	
	Volume Sanguin (microL)		Sang										
**Panel B**	100	**tube Rankl**	Rankl	CD25	CD38	CD27	IgD	CD24	IgM	CD3	CD5	CD19	**LBParo-PanelB**
vol (microL)			15	5	3,5	3,5	3,5	3,5	5	5	5	3,5	
	100	**tube isoRankl**	Iso	CD25	CD38	CD27	IgD	CD24	IgM	CD3	CD5	CD19	**LBParo-PanelB**
vol (microL)			3,5	5	3,5	3,5	3,5	3,5	5	5	5	3,5	
**Panel B1**	100	**tube Rankl**	Rankl	CD43	CD5	CD27	CD11b	CD24	CD20	CD3	CD69		**LBParo-PanelB1**
vol (microL)			15	10	5	5	5	5	5	5	10		
	100	**tube isoRankl**	Iso	CD43	CD5	CD27	CD11b	CD24	CD20	CD3	CD69		**LBParo-PanelB1**
vol (microL)			3,5	10	5	5	5	5	5	5	10		
**Panel T**	100	**tube Rankl**	Rankl	CD45RA	CD27	CD62L	CD38	CD3	CD4	CD8	CD25		**LBParo-PanelT**
vol (microL)			15	5	5	5	3	1	5	5	10		
	100	**tube isoRankl**	Iso	CD45RA	CD27	CD62L	CD38	CD3	CD4	CD8	CD25		**LBParo-PanelT**
vol (microL)			3,5	5	5	5	3	1	5	5	10		

Origines des anticorps utilisés

Panel B			Panel B1			Panel T		
**Marqueurs**	**Fluo**	**Localisation**	**Marqueurs**	**Fluo**	**Localisation**	**Marqueurs**	**Locai**	**Localisation**
**RANKL**	PE	PE254	**RANKL**	PE	PE254	**RANKL**	PE	PE254
**Iso IgG2b,k**	PE	PE25	**Iso IgG2b,k**	PE	PE25	**Iso IgG2b,k**	PE	PE25
**CD25**	FITC	FITC 25	**CD43**	FITC	FITC 43	**CD45RA**	FITC	FITC 45RA
**CD38**	PC5,5	PC5,5 38	**CD5**	PC5,5	PC5,5 5	**CD27**	PC5,5	PC5,5 27
**CD27**	PC7	PC7 27	**CD27**	PC7	PC7 27	**CD62L**	PC7	PC7 62L
**IgD**	APC	APC A5	**CD11b**	APC	APC 11b	**CD38**	APC	APC 38
**CD24**	AAF750	AAF750 24	**CD24**	AF750	AAF750 24	**CD3**	AAF750	AAF750 3
**IgM**	PB	PB B3	**CD3**	KO	KO 3	**CD4**	PB	PB 4
**CD3**	KO	KO 3	**CD20**	PB	PB 20	**CD8**	KO	KO 8
**CD5**	ECD	ECD 5	**CD69**	ECD	ECD 69	**CD25**	ECD	ECD 25
**CD19**	AAF700	AAF700 19						

Annexe 3
Protocole de prélèvement bactérien et de fluide gingival

En clinique :

- Explication de l'étude, de son intérêt et de ses objectifs
- Signatures des consentements (Consentement Biocollection OdontoBrest)
- Lors de l'examen clinique initial et lors de la réévaluation, les prélèvements seront effectués au niveau du l'intervention chirurgicale doit avoir lieu, ou au niveau des 2 sites suivants les plus profonds et d'un site sain. Les zones de prélèvements seront notées dans le fichier chartingparo.xlsx du patient
- Il faut d'abord prélever le fluide gingival et ensuite les bactéries, et non l'inverse
- Lorsque les prélèvements seront réalisés à la réévaluation, les tubes eppendorfs seront transportés au laboratoire et ils seront mis dans la même boite que celle de l'évaluation initiale

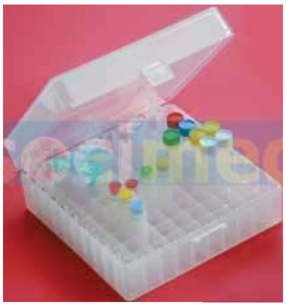

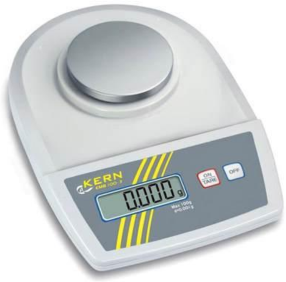

Matériel :
- Classeur biocollection OdontoBrest
- Fiche de contrôle et de prévention des incidents
- Marqueur indélébile
- Tubes eppendorf secs de 0,75 ml, stérilisées
- Pbs Tween à 0,05 %
- Pipette 20-200 µl
- Chronomètre réglé sur 30 secondes
- 2 boites de congélation : une pour le fluide gingival et l'autre pour le prélèvement bactérien
- Etiqueteuse
- Balance de précision, au 0,001g près (marque)
- Compresses stériles
- Cônes de papier stériles
- Ruban Adhésif (Scotch)

Prélèvement du fluide gingival

1- Préparer 4 tubes eppendorf : les peser avec le cône de papier qui servira au prélèvement.
2- Noter le poids de départ sur chartinparo.xlsx
3- Enlever le cône de papier stérile du tube
4- Isoler le site avec les cotons salivaires et compresses
5- Passer une compresse stérile sur la dent en cervical pour éliminer salive et biofilm en supragingival
6- Passer la soufflette sur la partie cervicale de la dent
7- Effectuer le prélèvement à l'aide du cône de papier stérile préparé pour le site, et ce durant 30 secondes
8- Mettre les cônes dans leur tube eppendorf respectif
9- Remplir les 4 tubes eppendorf avec 75 µl Pbs Tween 0,05 %
10- Mettre les tubes eppendorfs identifiés dans la boite de congélation (ou dans la boite initiale, au laboratoire)
11- Etiqueter la boite de congélation avec le numéro de la biocollection du patient

Prélèvement bactérien (dans la foulée du prélèvement gingival)

1- Insérer 2 cônes stériles côte à côte, attendre 30 secondes
2- Mettre le cône dans un tube eppendorf identifié (sans ajout de liquide)

Etiquetage des tubes :
Identification du tube :
- 2 derniers chiffres du numéro de biollection du patient
- F pour fluide gingival, B pour bactérien
- numéro de d'ordre du prélèvement
Recouvrir la plage d'écriture avec du ruban adhésif transparent (scotch)

Etiquetage des boites :
Identifier la boite de congélation avec le numéro de biocollection du patient et le type de prélèvement (F pour Fluide et B pour Bactérien)

***Ex pour BXADU0519690001**, sur le même site*
01 F 1 en initial
01 F 5 à la réévaluation

Dans la boite de congélation
XADU051969001F

3- Mettre les tubes eppendorfs identifiés dans la boite de congélation (ou dans la boite initiale, au laboratoire)
9- Etiqueter la boite de congélation avec le numéro de la biocollection du patient

Au laboratoire :

- Réception des échantillons
- Vérification de la fiche de contrôle de prévention des incidents
- Placement des boites de congélation au congélateur à -80°C
- Rangement de la fiche de contrôle de prévention des incidents dans le classeur approprié

Traitement des prélèvements :

- Envoie en groupe par transporteur spécialisé à Rennes pour les prélèvements bactériens et à Nantes pour les prélèvements de fluide gingival

Transport
Au moment de l'envoi, mettre les boites de congélation dans un sac plastique contenant du carboglace.
Enfouir le tout sous une quantité suffisante de carboglace dans une boîte en polystyrène expansé.
Sceller la boîte avec de l'adhésif.
Attention, utiliser un contenant qui respire (polystyrène) et n'est pas totalement hermétique, sinon risque d'explosion.
Avertir le laboratoire receveur, envoyer le prélèvement en début de semaine de façon à ce qu'il soit réceptionné et transféré dans un congélateur à -80°C
Envoyer par transport express type chronopost avec livraison en mains propres.

Annexe4
Protocole de gestion des données cliniques

Cheminement des données cliniques entre le centre de soins dentaires et le laboratoire d'immunologie

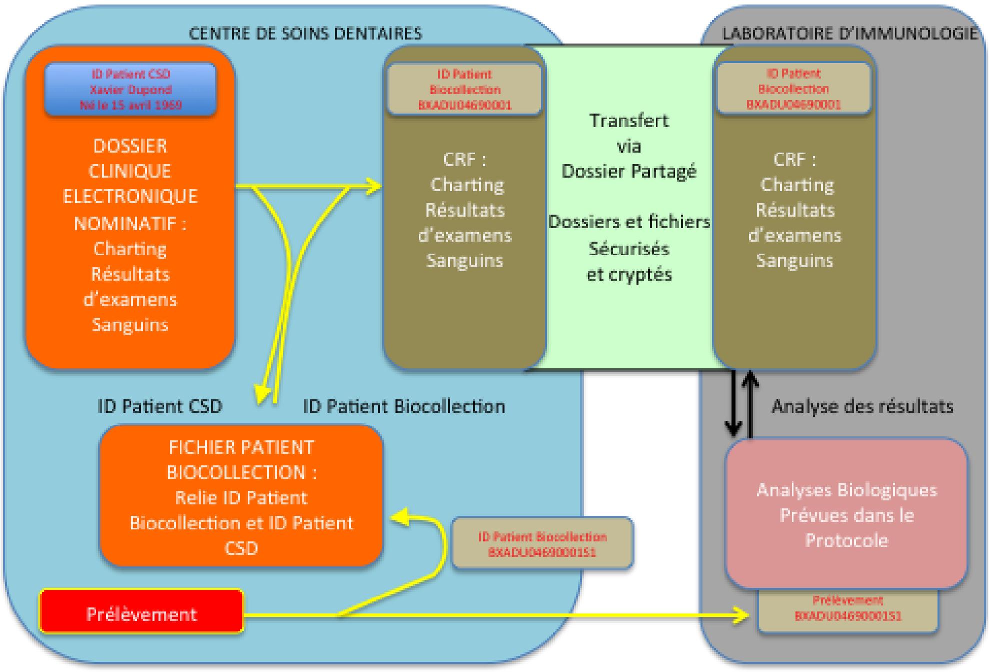

Construction du numéro de la biocollection : ex Xavier Dupond, né le 15 avril 1969

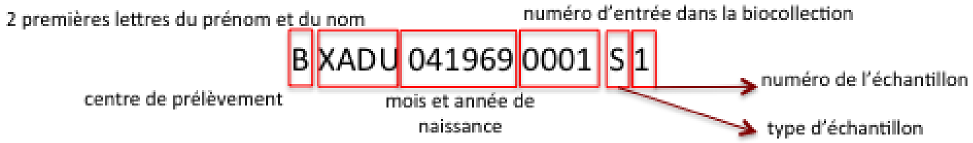

Au centre de soins dentaires :

Sur un ordinateur dédié du centre de soins dentaires, enregistrement des données nominatives du patient et des données non identifiables qui seront transmises au sein du réseau du CHU, via un dossier partagé. Remplissage des fichiers excel liés à l'étude : OdontoBrest-Patients.xlsx et chartingparo.xlsx

Dans un classeur dédié à l'étude :
- fiches papiers des protocoles de l'étude
- fiches vierges de contrôle et de prévention des incidents
- copies des consentements signés des patients

Au laboratoire d'immunologie :

Sur un ordinateur dédié du laboratoire d'immunologie, synchronisation via internet des données non identifiables du patient (chartingparo.xls) et enregistrement des résultats d'analyse biologique. Remplissage du fichier excel de recensement des échantillons reçus : OdontoBrest-LBParo.xlsx
Dans un classeur dédié à l'étude :

- fiches papier des protocoles de l'étude
- fiches de recensement des échantillons
- fiches remplies de contrôle et de prévention des incidents et rangées au fur et à mesure de la réception des échantillons

Type d'échantillon	Codes biocollection
Sang	S
Gencive	G
Fluide Gingival	F
Prélèvement Bactérien	B
Sérum/Plasma	P
Cellules Sanguines	CS
Salive	SA
Os	O

Annexe 5
Protocole de Prise en Charge des Patients en Parodontologie

1re étape : Observation clinique (en plusieurs séances de soins)

Déroulement de l'examen :

1- Examen clinique exo et endobuccal
2- Examen radiographique – remplissage du fichier chartingparo.xls –
3- Photographies
4- Révélation de plaque – Prise de l'indice d'Oleary – remplissage du fichier chartingparo.xls
5- Motivation au contrôle de plaque et prescription de matériel
6- Détartrage
7- Sondage parodontal – remplissage du fichier chartingparo.xls –
8- Prise de l'indice Gi en même temps que le sondage – remplir le fichier chartingparo.xls –
10- Faire rincer le patient à l'eau si prélèvements et sondage sont réalisés dans la même séance
11- Prélèvements bactériens et de fluide gingival
12- Diagnostic et pronostic parodontal
13- Prescription d'un bilan sanguin : NFS, VS, CRP, Plaquettes, Cholestérol total-HDL, Triglycérides, Glycémie à jeun

Matériel :
- sonde parodontale à pression constante (marque)
- miroir
- soufflette
- compresses stériles
- cônes de papier stériles
- eppendorfs stériles
- PBS TWEEN à 0,05%
- révélateur de plaque
- ordinateur avec les fichiers suivant :
  - charting électronique (chartingparo.xlsx)
  - fiches de recensement patient biocollection (OdontoBrest - Patients.xlsx)
- fiche vierge de contrôle et de prévention des incidents

2e étape : Inclusion du patient

Si le patient rentre dans les critères d'inclusion à l'étude.
1- Présentation de l'étude au patient et de la biocollection
2- Signature de la fiche de consentement du patient si acceptation
3- Détermination d'un numéro de biocollection et recensement dans le fichier OdontoBrest-Patients.xlsx

3e étape : Plan de traitement

1- Détermination du plan de traitement global et parodontal
2- Prise des rendez-vous de soins et du rendez-vous pour la prise de sang auprès de l'IDE

4e étape : Actions thérapeutiques

En fonction du plan de traitement :

Si pas d'extraction prévue : réalisation de la thérapeutique initiale
Si extractions prévues : prélèvement gingival durant l'extraction et réalisation de la thérapeutique initiale

Lors de la réévaluation parodontale :

Nouvel examen parodontal complet et prélèvements
Décision chirurgicale : extraction dentaire, chirurgie d'assainissement
Prescription d'un bilan sanguin : NFS, VS, CRP, Plaquettes et bilan de l'Hémostase (TCA, TP)

Réalisation des thérapeutiques chirurgicales décidées et prélèvement gingival.

Inclusion
- âgé de plus de 18 ans
- nécessitant une chirurgie buccale de type
  - extraction dentaire
  - chirurgie parodontale
  - chirurgie mucogingivale
- en bonne santé générale
- avec une suspicion de troubles de l'hémostase ou de la présence d'un diabète

Non-inclusion - Exclusion
- âgé de moins de 18 ans
- prise d'antibiotiques dans les 3 mois qui précèdent l'analyse
- femme enceinte
- pathologie systémique, notamment une pathologie inflammatoire chronique
- caries non traitées
- en cours de traitement orthodontique

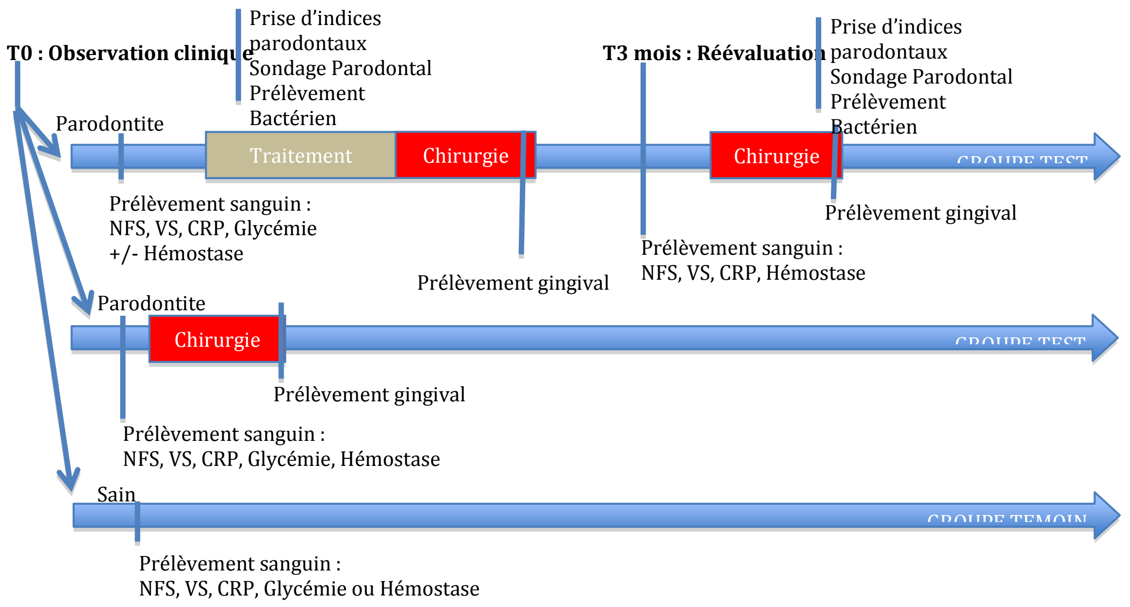

Protocole de Prise en Charge des Patients en Parodontologie

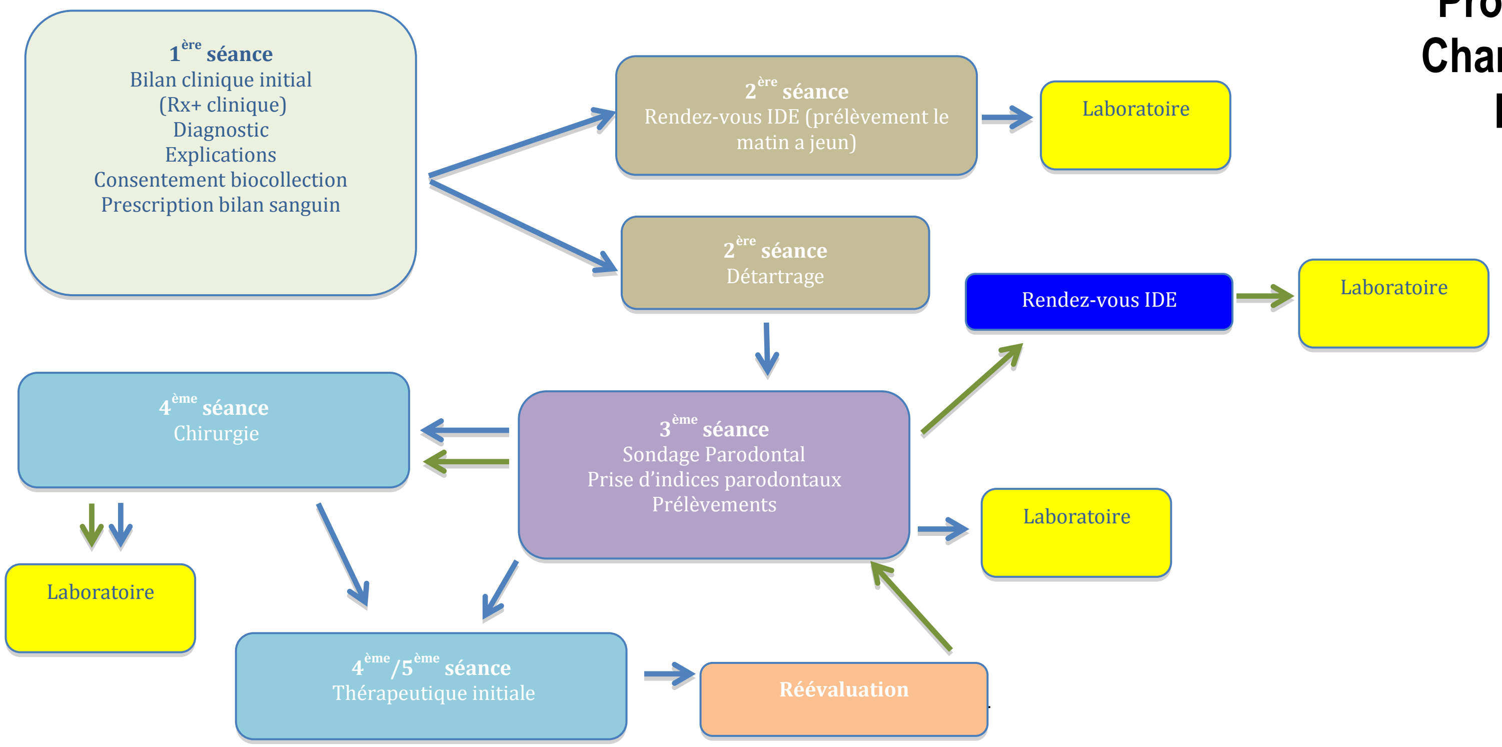

Praticiens :
Camille FRAMERY
Dr Julien DEMOERSMAN
Dr Juliette COAT
Dr Claire HENRY
Dr Guy LETOUX
Dr Cédric LANSONNNEUR
Dr Sylvie BOISRAME-GASTRIN
Dr Julien DEMOERSMAN
Pr Jacques-Olivier PERS

Version : 17/04/2015

Annexe 6

Fiche de contrôle et de prévention des incidents

Date : …………………………………………………………………………………..

N° d'identification dans la biocollection OdontoBrest
|__|__|-|__|__|.- |__|__||__|__||__|__| - |__|__||__|__|
(2 1ères lettres du nom et prénom) - (mois et année de naissance) – N° inclusion

Type d'échantillon : *Sang* ☐ Gencive ☐

Fluide gingival ☐ Bactéries ☐

Nombre d'échantillons : ………………………………………………………………

A remplir par l'investigateur clinique :
Nom : ……………………………………………………………………………………………………..

Etapes :

- ☐ Explication de l'étude
- ☐ Signature du consentement par le patient
- ☐ Attribution d'un numéro de biocollection au patient
- ☐ Remplissage du fichier OdontoBrest-Patients
- ☐ Examen Clinique, Prise d'indice et Sondage
- ☐ Remplissage du fichier Chartingparo
- ☐ Prescription examen sanguin

Vérification des étiquetages et des rangements :

- ☐ Tubes Eppendorfs correctement rangés et étiquetés
- ☐ Boites de congélations correctement étiquetés
- ☐ Tubes de sang correctement étiquetés
- ☐ Sachet avec échantillon gingival correctement étiqueté

Commentaires et report d'incidents :
……
……
……
……
……
……

A remplir par le réceptionnaire au laboratoire :
Nom : ……………………………………………………………………………………………………..

Vérification des étiquetages et des rangements :

- ☐ Tubes Eppendorfs correctement rangés et étiquetés
- ☐ Boites de congélations correctement étiquetées
- ☐ Tubes de sang correctement étiquetés
- ☐ Sachet avec échantillon gingival correctement étiqueté

Réception :

- ☐ Rangement des boites de congélations dans le congélateur -80°C
  - localisation du congélateur : …………………………
  - étagère de stockage :…………………………………
- ☐ Traitement des échantillons de sang et de gencive

Commentaires et report d'incidents :
……
……
……
……
……
……

A remplir par l'investigateur au laboratoire :
(La fiche doit suivre l'échantillon si transport vers un autre centre)
Nom : ....

Vérification des étiquetages et des rangements :

☐ Tubes Eppendorfs correctement rangés et étiquetés
☐ Boites de congélations correctement étiquetées
☐ Tubes de sang correctement étiquetés
☐ Sachet avec échantillon gingival correctement étiqueté
☐ Tube de sérum correctement étiqueté

Commentaires et report d'incidents durant l'analyse des échantillons :

....
....
....
....
....
....
....
....
....
....
....
....
....
....
....
....
....
....
....
....
....
....
....
....
....
....
....
....
....
....
....
....
....
....
....
....
....
....
....

Annexe 7
Fiche d'inclusion des cas et des témoins

	Parodontite et inflammation :<br>Approche clinique et biologique du rôle des lymphocytes B<br>LBPARO<br><br>Identification du Cas |__|__|-|__|__|.- |__|__||__|__||__|__| - |__|__||__|__| (2 ères lettres du nom et prénom) - (mois et année de naissance) – N° inclusion

INCLUSION DES CAS

Date d'inclusion |__|__| |__|__| |__|__|

CRITRES D'INCLUSION	OUI	NON
Age ≥ 18 ans		
Souffrant d'une parodontite chronique ou agressive,		
Nécessitant une chirurgie type parodontale		
Avec une suspicion de troubles de l'hémostase ou de la présence d'un diabète		
En bonne santé général		
Ayant signé leur consentement<br>- **Date |__|__| |__|__| |__|__|**		

Si une ou plusieurs cases « NON » sont cochées le patient n'est pas inclus

CRITRES DE NON INCLUSION	OUI	NON
Mineur		
Patient ayant pris des antibiotiques dans les 3 mois précédents l'étude		
Patient présentant une pathologie systémique notamment pathologie inflammatoire chronique		
Femme enceinte		
Patient en cours de traitement orthodontique		

Si une ou plusieurs cases « OUI » sont cochées le patient n'est pas inclus

Date : |__|__| |__|__| |__|__|
Nom de l'investigateur :
Signature

INCLUSION DES TEMOINS

Date d'inclusion |__|__| |__|__| |__|__|

CRITRES D'INCLUSION	OUI	NON
Age ≥ 18 ans		
Nécessitant une extraction dentaire ou une chirurgie mucogingivale		
Ayant une suspicion de troubles de l'hémostase ou de la présence d'un diabète		
En bonne santé général		
Ayant signé leur consentement<br>- **Date |__|__| |__|__| |__|__|**		

Si une ou plusieurs cases « NON » sont cochées le patient n'est pas inclus

CRITRES DE NON INCLUSION	OUI	NON
Mineur		
Souffrant d'une parodontite chronique ou agressive,		
- prise d'antibiotiques dans les 3 mois qui précèdent l'étude		
Patient présentant une pathologie systémique notamment pathologie inflammatoire chronique		
Femme enceinte		
Patient en cours de traitement orthodontique		

Si une ou plusieurs cases « OUI » sont cochées le patient n'est pas inclus

Date : |__|__| |__|__| |__|__|
Nom de l'investigateur :
Signature

Annexe 8
Formulaire de consentement
