# Supplementary material for: B cell subset distribution is altered in patients with severe periodontitis
Source: PLoS One. 2018 Feb 15;13(2):e0192986. doi: 10.1371/journal.pone.0192986 (PMC5814041; doi:10.1371/journal.pone.0192986)
Supplement: S2 Fig — (PDF) [file pone.0192986.s003.pdf]

We updated the design of this site on December 18, 2017. Learn more.

U.S. National Library of Medicine
ClinicalTrials.gov

Trial record **1 of 1** for: NCT02833285

Previous Study | Return to List | Next Study

B Cell Functions in Periodontitis (LBPARO)

The safety and scientific validity of this study is the responsibility of the study sponsor and investigators. Listing a study does not mean it has been evaluated by the U.S. Federal Government. Know the risks and potential benefits of clinical studies and talk to your health care provider before participating. Read our disclaimer for details.

ClinicalTrials.gov Identifier:
NCT02833285

Recruitment Status :
Recruiting
First Posted : July 14, 2016
Last Update Posted :
February 1, 2017

See **Contacts and Locations**

Sponsor:
University Hospital, Brest

Information provided by (Responsible Party):
University Hospital, Brest

Study Details | **Tabular View** | **No Results Posted** | Disclaimer | How to Read a Study Record

Study Description

Go to

Brief Summary:

The inflammatory response involves many players from the immune response, including B lymphocytes. These cells are responsible for the synthesis of immunoglobulins in response to the presence of an antigen. They are characteristic of chronic inflammation. There are several subsets of B cells characterized by specific membrane markers. Once activated, these cells express many factors contributing to tissue destruction seen in periodontitis and particularly in osteoclastogenesis

(receptor activator of nuclear factor kappa-B ligand, tumor necrosis factor, interleukin-6, macrophage inflammatory protein-1α and Monocyte Chemoattractant Protein-3).

During the establishment of a periodontal disease, an important inflammatory infiltrate is observed in the gum. This infiltrate is characterized by the presence of many B lymphocytes. B cell subsets in the blood and the gum of patients with periodontitis have been little studied. However, the number of autoreactive B cells (cluster of differentiation (CD)19+, CD5+) has been reported to be higher in the blood of patients with periodontal disease. In the gum, the rate of B and T cells increases with the level of inflammation and is correlated with the severity of the inflammatory process. Activation of B cells is a prerequisite for the progression of gingivitis to periodontitis. B cell distribution could then be an indicator of disease progression, but also allow to study the response to treatment.

The aim of this pilot study is to characterize B cell subsets in the blood and the gum of patients with periodontitis, according to disease activity. Analysis of B cells in the blood could highlight the association of a particular subpopulation with aggressive periodontal disease and evidence a particular biological profile of the host response. The investigators also wish to observe the evolution of this phenotype following an unconventional surgical therapy.

This study would better understand the pathogenesis of periodontal disease and refine the diagnosis, prognosis and treatment of periodontitis, and thus participate in the development of personalized medicine. Biological monitoring of therapeutic effects may be initiated and allow more effectively prevent recurrence.

Condition or disease
Chronic Periodontitis
Aggressive Periodontitis

Study Design

Go to

Study Type :	Observational
Estimated Enrollment :	60 participants
Observational Model:	Case-Control
Time Perspective:	Cross-Sectional
Official Title:	Periodontitis and Inflammation : Biological and Clinical Approach of B Cell Role.
Study Start Date :	May 2015
Estimated Primary Completion Date :	September 2018
Estimated Study Completion Date :	September 2018

Groups and Cohorts

Go to

Outcome Measures

Go to

Primary Outcome Measures :

1. Phenotype of B cells in the blood and gums [Time Frame: 3 months]

Biospecimen Retention: Samples With DNA
gingiva biopsy gingival fluid blood

Eligibility Criteria

Go to

Information from the National Library of Medicine NIH NLM

Choosing to participate in a study is an important personal decision. Talk with your doctor and family members or friends about deciding to join a study. To learn more about this study, you or your doctor may contact the study research staff using the contacts provided below. For general information, Learn About Clinical Studies.

Ages Eligible for Study:	18 Years to 75 Years (Adult, Senior)
Sexes Eligible for Study:	All
Accepts Healthy Volunteers:	Yes
Sampling Method:	Probability Sample

Study Population

Male or female from 18 to 75 years old before periodontal treatment

Criteria

Inclusion Criteria:

- Requiring periodontal surgery
- In good health
- Having signed consent

Exclusion Criteria:

- Minor
- Patient having taken antibiotics in the previous 3 months
- Patients with systemic diseases including chronic inflammatory disease
- Pregnancy
- orthodontic treatment ongoing

Contacts and Locations

Go to

Information from the National Library of Medicine NIH NLM

To learn more about this study, you or your doctor may contact the study research staff using the contact information provided by the sponsor.

Please refer to this study by its ClinicalTrials.gov identifier (NCT number): ***NCT02833285***

Contacts

Contact: PERS Jacques-Olivier 02 98 32 33 84 pers@univ-brest.fr

Locations

France

CHRU de Brest **Recruiting**
Brest, France, 29609
Contact: PERS Jacques-Olivier

Sponsors and Collaborators

University Hospital, Brest

Investigators

Principal Investigator: PERS Jacques-Olivier University Hospital of Brest

More Information

Go to

Responsible Party:	University Hospital, Brest
ClinicalTrials.gov Identifier:	NCT02833285 History of Changes
Other Study ID Numbers:	LBPARO
First Posted:	July 14, 2016 Key Record Dates
Last Update Posted:	February 1, 2017
Last Verified:	January 2017

Individual Participant Data (IPD) Sharing Statement:
Plan to Share IPD: No

Additional relevant MeSH terms:

Periodontitis	Periodontal Diseases
Chronic Periodontitis	Mouth Diseases
Aggressive Periodontitis	Stomatognathic Diseases
