# Supplementary material for: B cell subset distribution is altered in patients with severe periodontitis
Source: PLoS One. 2018 Feb 15;13(2):e0192986. doi: 10.1371/journal.pone.0192986 (PMC5814041; doi:10.1371/journal.pone.0192986)
Supplement: S3 Fig — (PDF) [file pone.0192986.s004.pdf]

EXTRAIT DU PROCES VERBAL DU COMITE D'ETHIQUE

SEANCE DU 09 Avril 2015

Sous la présidence par intérim du Docteur Véronique AMICE

COMITE D'ETHIQUE

Présidente par intérim

Dr Véronique Amice
Biologie de la reproduction
Veronique.amice@chu-brest.fr

Vice Présidente

Mme Patricia Le Gall
Infirmière en psychiatrie
Patricia.loisel@chu-brest.fr

Secrétaire

M. Rémi BRAJEUL
Directeur Adjoint
Remi.brajeul@chu-brest.fr

Secrétariat
02 98 22 39 66

Titre du protocole
« Parodontite et inflammation : approche clinique et biologique du rôle du lymphocyte B – LBParo ».

Protocole version V1.4 du 17/04/15, consentement institutionnel proposé dans le cadre de la déclaration de la biocollection OdontoBrest (DC 2014-2158).

Ce protocole est soumis par le Pr Jacques-Olivier Pers du laboratoire d'Immunologie du CHRU de Brest.

Justification de l'étude :
Les maladies parodontales, gingivites et parodontites, sont des maladies infectieuses d'origine bactérienne à manifestations inflammatoires qui affectent le parodonte. Elles se traduisent par des lésions inflammatoires, confinées à la gencive dans le cas des gingivites, ou étendues à l'ensemble des tissus parodontaux dans le cas des parodontites. Elles sont alors responsables d'une perte d'attache parodontale pouvant aboutir à la perte de l'organe dentaire et d'une bactériémie à distance.
La réaction inflammatoire, élément central de la maladie parodontale, fait intervenir de nombreux acteurs de la réponse immunitaire, notamment les lymphocytes B (LB). Ce sont les cellules de l'immunité humorale, responsables de la synthèse des immunoglobulines ou anticorps face à la présence d'un antigène. Elles sont caractéristiques d'une inflammation chronique. Il existe plusieurs classes de lymphocytes B caractérisés par des antigènes de surface spécifique (marqueurs membranaires). Une fois activée, ces cellules expriment de nombreux facteurs impliqués, notamment dans l'ostéoclastogénèse (RANKL, TNFα, IL-6, MIP-1α et MCP-3) et participent ainsi aux destructions tissulaires observées dans la parodontite.
Lors de la mise en place d'une atteinte parodontale, un important infiltrat inflammatoire est observé au sein de la gencive. Cet infiltrat est caractérisé par la présence de nombreux lymphocytes B issus du sang par diapédèse. Les sous-populations de LB présentes dans le sang et la gencive n'ont été que peu étudiées. Cependant, le nombre de LB autoréactifs (CD19+, CD5+) a été rapporté comme étant plus élevé dans le sang des patients souffrant de maladie parodontale. Dans la gencive, le taux des lymphocytes B et T augmente en fonction du niveau de l'inflammation et est corrélé avec la sévérité du processus inflammatoire. L'activation des LB est un préalable à l'évolution de la gingivite vers la parodontite ce qui suggère qu'il doit exister des lésions stables caractérisées par des LB au repos. Le LB pourrait être alors un indicateur de l'évolution de la maladie, mais aussi permettre d'étudier la réponse au traitement
L'objectif de cette étude pilote est de caractériser les sous-populations des LB présentes chez un patient souffrant d'une parodontite, en fonction du type de maladie parodontale diagnostiqué et de son niveau d'activité, défini sur le plan biologique (MMP8, RANK-L). Cette étude permettra de déterminer si les LB présents ont un phénotype de LB "transitionnel", de

LB "immature", de LB "mature" ou de LB "mémoire" à la fois dans le sang et dans des biopsies gingivales. L'analyse des LB dans le sang pourrait permettre de mettre en évidence l'association d'une sous-population particulière avec l'agressivité de la maladie parodontale et un profil biologique particulier de la réponse de l'hôte, donc du type de parodontite.
Cette étude permettrait donc de mieux comprendre la physiopathologie des maladies parodontales, d'en affiner le diagnostic, le pronostic et le traitement d'une parodontite et ainsi participer à la mise en place d'une médecine personnalisée. Un suivi biologique des effets des thérapeutiques pourrait être instauré et permettre de prévenir plus efficacement la récidive.

Population concernée :
Cas : Patients souffrant de maladie parodontale chronique ou agressive.
Témoins : Patients ne souffrant pas de parodontite et devant subir une chirurgie dentaire incluant la réalisation d'une analyse sanguine.

Objectif principal :
Analyse du phénotype des lymphocytes B au niveau du sang et de la gencive des patients souffrant de différents types de maladies parodontales

Critère de jugement principal :
Analyse Phénotypique des lymphocytes B dans le sang et les gencives

Méthodologie :
Etude physiopathologique prospective monocentrique sur données clinico-biologiques
Analyse sur plusieurs groupes de parodontite en fonction de critères cliniques (agressive, chronique) et d'activité vs groupe contrôle sans .

Nombre de sujets prévus :
15 patients avec une parodontite chronique 15 patients avec une parodontite agressive, 30 témoins.

Déroulement de l'étude :
Le protocole est exposé au patient venant consulter au centre de soins dentaire du CHRU de Brest et chez qui un diagnostic de parodontite, ou non, est réalisé, nécessitant un prélèvement sanguin en vue de s'assurer de l'absence d'un diabète ou de troubles de l'hémostase. Son accord de participation à l'étude est alors recherché pour son inclusion (consentement) soit en en tant que « cas-parodontite" soit en tant que 'Témoin'.
Les prélèvements sont alors réalisés dans le cadre du parcours de soin du patient dans un but de diagnostic ou de dépistage (diabète et troubles de l'hémostase).
Le suivi de la parodontite ne sera pas modifié par le protocole. la réévaluation aura lieu à 3 mois en consultation de routine pour évaluer le résultat du traitement parodontal non chirurgical. Lors de cette consultation, où une nouvelle observation clinique sera réalisée, de nouveaux prélèvements sanguins et gingivaux pourront êtres obtenus si une intervention de chirurgie buccale était indiquée.

L'étude ne modifiera donc en rien les habitudes du service ni les modalités de prise en charge

Conclusion :
Il s'agit donc d'une étude observationnelle, qui ne relève pas, en l'état, de la loi sur la recherche biomédicale.

Avis
Après en avoir délibéré, et à l'unanimité, le comité d'éthique fait savoir qu'il ne soulève aucune objection sous l'angle de l'éthique à l'étude telle que présentée.
Ce projet devra faire l'objet d'une déclaration normale à la CNIL

Pour extrait conforme, le Secrétaire du Comité d'Ethique

Rémi BRAJEUL
